# Deep learning enables image-based tree counting, crown segmentation, and height prediction at national scale

**DOI:** 10.1093/pnasnexus/pgad076

**Published:** 2023-03-09

**Authors:** Sizhuo Li, Martin Brandt, Rasmus Fensholt, Ankit Kariryaa, Christian Igel, Fabian Gieseke, Thomas Nord-Larsen, Stefan Oehmcke, Ask Holm Carlsen, Samuli Junttila, Xiaoye Tong, Alexandre d’Aspremont, Philippe Ciais

**Affiliations:** Department of Geosciences and Natural Resource Management, University of Copenhagen, Copenhagen 1350, Denmark; Département Sciences de la terre et de l'univers, espace, Université Paris-Saclay, Gif-sur-Yvette 91190, France; Department of Geosciences and Natural Resource Management, University of Copenhagen, Copenhagen 1350, Denmark; Department of Geosciences and Natural Resource Management, University of Copenhagen, Copenhagen 1350, Denmark; Department of Computer Science, University of Copenhagen, Copenhagen 2100, Denmark; Department of Computer Science, University of Copenhagen, Copenhagen 2100, Denmark; Department of Computer Science, University of Copenhagen, Copenhagen 2100, Denmark; Department of Information Systems, University of Münster, Münster 48149, Germany; Department of Geosciences and Natural Resource Management, University of Copenhagen, Copenhagen 1350, Denmark; Department of Computer Science, University of Copenhagen, Copenhagen 2100, Denmark; Department of Earth Observations, The Danish Agency for Data Supply and Infrastructure, Copenhagen 2400, Denmark; Department of Forest Sciences, University of Eastern Finland, Joensuu 80101, Finland; Department of Geosciences and Natural Resource Management, University of Copenhagen, Copenhagen 1350, Denmark; Department of Computer Science, École Normale Supérieure, Paris 75230, France; Laboratoire des Sciences du Climat et de l'Environnement, CEA, CNRS, UVSQ, Université Paris-Saclay, Gif-sur-Yvette 91190, France

**Keywords:** deep learning, remote sensing, individual trees, forest inventory

## Abstract

Sustainable tree resource management is the key to mitigating climate warming, fostering a green economy, and protecting valuable habitats. Detailed knowledge about tree resources is a prerequisite for such management but is conventionally based on plot-scale data, which often neglects trees outside forests. Here, we present a deep learning-based framework that provides location, crown area, and height for individual overstory trees from aerial images at country scale. We apply the framework on data covering Denmark and show that large trees (stem diameter >10 cm) can be identified with a low bias (12.5%) and that trees outside forests contribute to 30% of the total tree cover, which is typically unrecognized in national inventories. The bias is high (46.6%) when our results are evaluated against all trees taller than 1.3 m, which involve undetectable small or understory trees. Furthermore, we demonstrate that only marginal effort is needed to transfer our framework to data from Finland, despite markedly dissimilar data sources. Our work lays the foundation for digitalized national databases, where large trees are spatially traceable and manageable.

**Significance Statement**

We present a framework based on state-of-the-art computer science methods that can be used to establish national databases featuring location, crown area, and height for large or overstory trees, both inside and outside forests. We apply the model on aerial images from Denmark and Finland, yet it can be transferred and adapted to different data sets covering diverse landscape types. Our framework can assist current national forest inventories, thereby becoming an important complementary data source to field studies, supporting digitalized forest management and policy planning for a green transition.

## Introduction

Climate change and rapid losses of forest habitats and biodiversity are the major environmental challenges of the 21st century (1, 2). Sustainable forest management can mitigate these crises by building carbon stocks, providing materials for a green economy, and developing habitats representing the most important reservoir for biodiversity in the world (3, 4). Consequently, policies addressing climate change mitigation and adaptation, sustainable wood production, and biodiversity must rely on timely, detailed, and reliable information on the state and development of tree resources and habitats.

Detailed knowledge of forests at regional and national scales is commonly obtained from inventories such as the national forest inventories (NFI). Here, variables such as tree diameter, height, species, growth, and mortality are recorded during repeated census on a representative sample of widely distributed plots (5–8). Inventories provide essential information on forest biomass stocks used for climate treaties and carbon accounting but are time-consuming, labor-intensive, and limited to plot scale, and the methods, and degree to which monitoring of trees outside forests is conducted, vary substantially across countries (9, 10). Comprehensive information on forests, such as forest cover (11), structure (12), resources (13, 14), phenology (15, 16), disturbances (17), and diversity (14, 18, 19) at national scale, is commonly derived from remote sensing data, often combined with inventory measurements (20). Satellite-based monitoring of forests based on readily available satellite data with a spatial resolution down to 10 m enables low-cost (21) and wall-to-wall assessments that can be rapidly repeated at a high temporal frequency and a large scale (11). However, results at this spatial resolution are not easy to interpret, and changes in the satellite data processing methods between older and newer sensors can lead to bias in time series. For example, recent declines in tree cover and increases in forest harvest in Europe inferred from Landsat data were shown to be an artifact from the processing algorithm (22, 23). Overall, average forest characteristics based on spatially aggregated attributes, such as height or volume proxies, ignore the diversity of individual trees, i.e. the fact that trees generally have variable height and crown sizes (24–26). Since the provisioning of ecosystem services, such as forest resources and habitats, as well as forest management, is closely related to individual trees (27, 28), new methods are required to characterize their distribution and size across large spatial areas.

In addition, most NFI and satellite-based studies do not include systematic assessments of trees outside forests (9). Previous studies have shown that trees outside forests, such as in urban or agricultural landscape types, can constitute a considerable part of the national wood resources (29–32) and provide a variety of ecosystem services (9, 33). Measuring trees outside forests from space-borne sensors is challenging, because the crown size of an isolated tree is typically smaller than the spatial resolution of readily available satellite images (34, 35), and the heterogenous spatial distribution is difficult to assess with field plots (9).

Current state-of-the-art approaches based on airborne light detection and ranging (LiDAR) data (36–38) have the potential to meet the requirements to support management and conservation policies. LiDAR data have been successfully applied to derive essential variables such as tree cover (39), stem volume (40, 41), and carbon stocks (42). However, the scanning is often performed on a snapshot basis during irregular campaigns, which can be costly for a national coverage. Recent advances in computer vision have shown that single trees can also be mapped using submeter resolution (50 cm) satellite, aerial, or drone imagery (43–49), but this has rarely been conducted at national scale, where challenges could arise from different landscape types, tree characteristics, or image preprocessing. An exception is the study by Brandt et al. (50), who mapped billions of individual trees and shrubs in the sub-Saharan desert and Sahelian savanna landscape types. Yet, it has been questioned whether the approach designed for mapping single trees growing in isolation in dry areas could be transferred to the European forest setting, where closed forests prevail. Moreover, Brandt et al. (50) did not embark upon assessing the height of individual trees, which is an essential variable for estimating biomass and carbon stocks (51–53). Submeter resolution aerial imagery is publicly available for many European countries and is frequently updated. Identifying overstory tree crowns from these images is potentially possible for the human eye, and deep learning methods have achieved great success in solving similar problems, including microscopy cell segmentation (54–56), scene labeling (57, 58), and human crowd counting (59, 60). However, the capacity of deep learning to segment and count tree crowns has rarely been verified for closed forests at national scales, and it remains uncertain to which extent small or understory trees are missed. Furthermore, inferring height, i.e. structural information, from merely optical images remains an important yet challenging problem (61), especially at the level of individual trees (62, 63).

**Fig. 1.**
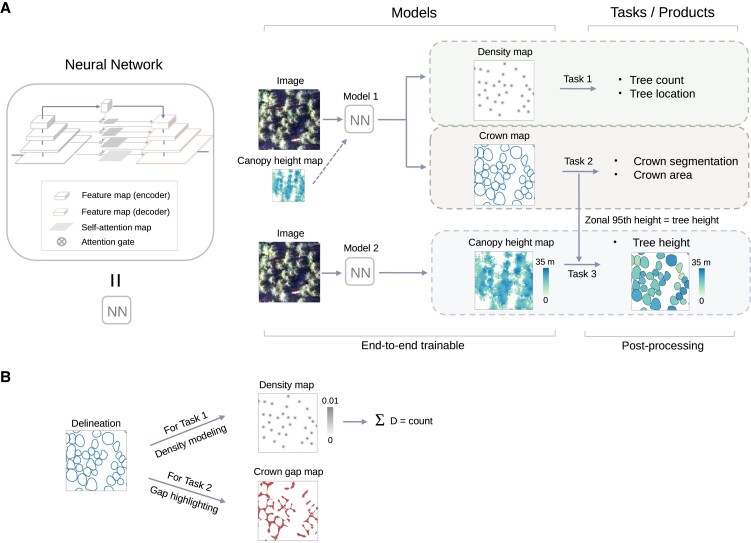
Overview of the framework used to count individual trees and predict their crown area and height. A) Deep learning-based framework for individual tree counting, crown segmentation, and height prediction. Spatial locations of individual trees are incorporated in the tree density maps and the crown segmentation maps. The canopy height map (CHM) derived from LiDAR data provides pixel-wise height information, which, when available for a specific study area, can optionally be used as an additional input band for the individual tree counting and crown segmentation tasks. B) Data preparation and modeling for tree counting and crown segmentation. The manually delineated individual tree crowns are modeled as density maps for the counting task by extracting the polygon centroids. The gaps between adjacent crowns are highlighted for the separation of individual tree crowns during the training phase.

Here, we aim at testing the ability of convolutional neural networks (64, 65) to produce a national airborne tree inventory, including attributes on individual trees, such as location, crown area, and tree height (Fig. 1) from images. We apply the framework on aerial images covering Denmark (2018) at a spatial resolution of 20 cm and generate countrywide maps on the number, location, crown size, and height of trees in diverse landscape types, including dense forests, open fields, and urban areas. We aim at mapping all woody plants visible from an aerial image perspective and use NFI data to quantify undetected large trees (stem diameter >10 cm) and undetected small trees (stem diameter <10 cm and height >1.3 m), which are likely from understory in forests. We further test our models on aerial images from Finland, France, and the United States of America and demonstrate the adaptability of our method by transferring model weights learned from data covering Denmark to data covering Finland. A successful transfer implies that, once a model is trained, it can be adapted to different data sets, and height estimation of trees can be obtained without further need for LiDAR data.

## Results

**Fig. 2.**
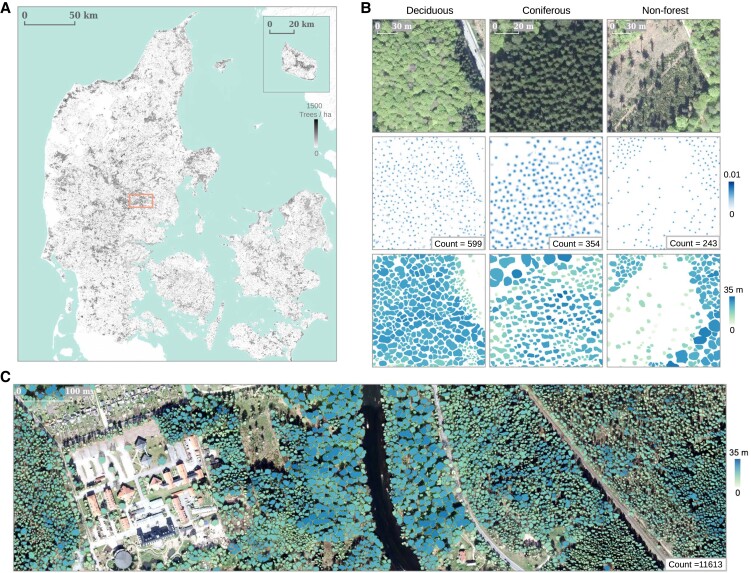
Example products from the proposed framework. A) Wall-to-wall tree count prediction for Denmark. B) Detailed examples showing the individual tree counting (second row), crown segmentation (third row), and height prediction (third row) from three major types of landscapes (deciduous forest, coniferous forest, and nonforest). C) Large-scale individual tree crown segmentation results colored by height predictions. Examples in B) and C) were sampled from the region indicated by the square shape in A).

The proposed framework involves two separate models addressing three localization and characterization tasks of individual trees (Fig. 1). The first model solves the tree counting and crown segmentation tasks jointly from multiband aerial images and a canopy height map derived from LiDAR data. Including a canopy height map is not pivotal but leads to marginally improved results (Table S1 and Fig. S1). The second model uses LiDAR data as training data and predicts canopy heights from multiband aerial images. The predicted canopy heights are further combined with the crown segmentation results to obtain the height per tree, which we define as the 95th percentile height within each predicted tree crown. Example products from the proposed framework are shown in Fig. 2.

### Multitask deep learning enables simultaneous tree counting and crown segmentation

We established a multitask deep learning-based network for jointly solving the individual tree counting (Task 1 in Fig. 1) and crown segmentation tasks (Task 2 in Fig. 1) from 2D imagery for both forest and nonforest trees (66). As input data, we used RGB and near-infrared (NIR) aerial images at 20-cm resolution from summer 2018 and a canopy height map projected from airborne LiDAR data at 40-cm resolution. Note that the input data can be aerial images with different spectral band compositions and the inclusion of a height map from LiDAR is optional. Performance comparison between models trained with different sources of input data can be found in Table S1 and Fig. S1. As target output references, 21,787 individual tree crowns from different forest and nonforest landscape types were manually delineated by visually inspecting the aerial images without any semiautomatic assistance (Fig. S2). We labeled trees with identifiable shadows, and adjoining crowns were delineated as separate individual segments. We observed that forest trees, particularly for a dense cover of deciduous trees, tend to naturally exhibit a clustered spatial pattern, thus making it challenging to delineate or count individual tree crowns. To separate adjoining tree crowns, the gaps in between neighboring crowns were fed into the model along with the crown delineations to enforce the model to be attentive to the crown boundaries (Fig. 1B) (50). To count trees, we used a density estimation approach (60), where each tree crown was represented by a small sample point located at its centroid on the density map, and the total tree count in an image of arbitrary size was equal to the integral of the density map (Fig. 1b). The model was primarily adapted from the U-Net architecture (64), with two output branches for the counting and crown segmentation tasks. More details about the training process can be found in the Methods section. For model evaluation as described below, we created an independent test data set with 2,679 annotated tree crowns in randomly selected plots distributed all over Denmark (Fig. S2c). Here, we evaluated the performance against manual delineations that the model has never been exposed to during training or model selection to justify the capacity of the deep learning network. We present the field evaluation against inventory data in a following section.

**Fig. 3.**
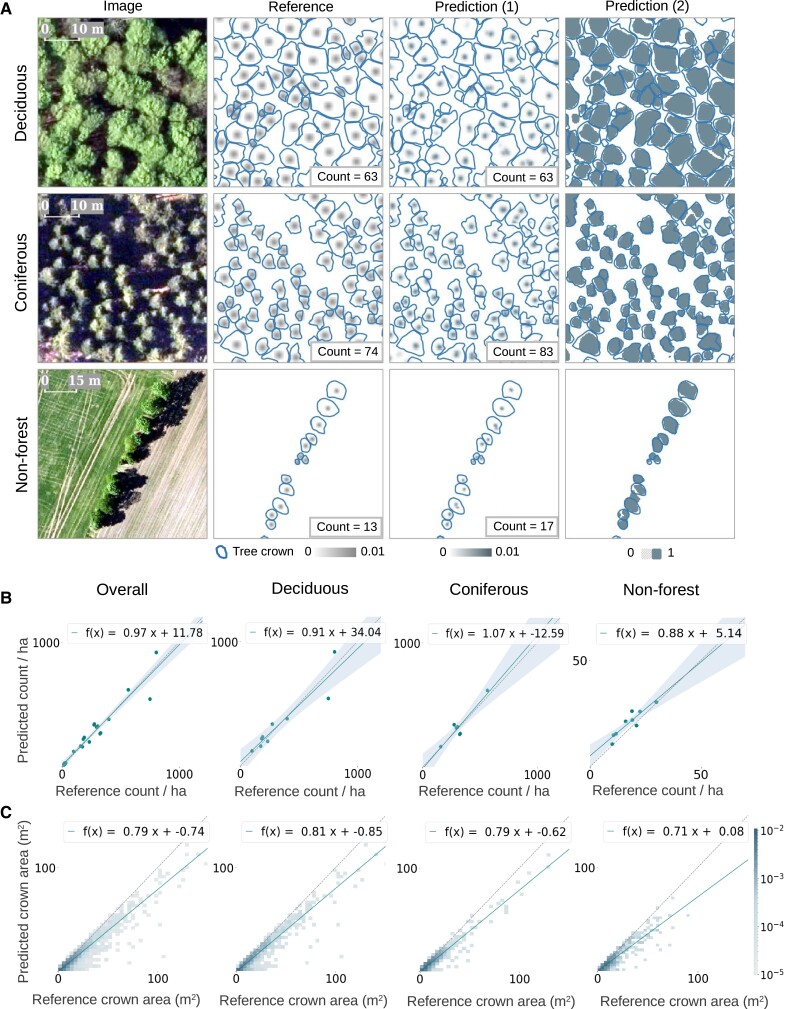
Individual tree counting and crown segmentation performance on the test data set. A) Examples from three different forest/landscape types: deciduous forest, coniferous forest, and nonforest areas. Reference shows the target labels, including the manual crown delineations (solid lines) and the Gaussian-blurred crown centroids (center dots). Prediction (1) shows the counting by density estimation results, and prediction (2) shows the crown segmentation results, both overlaid with the manual delineations (solid lines). B) Evaluation of the tree counts from prediction (1), grouped in respective landscape types and rescaled to tree counts per hectare. Here, each scatter point in the plots represents a sampling image of varying sizes ranging between 0.07 and 6.7 ha (see details in the main text). The regression lines are shown in solid and the identity lines are shown in dotted. C) Evaluation of the tree crown area predictions from prediction (2) at individual tree-level accuracy.

The independent test data set covered three major types of landscapes (66), including high-density deciduous forests (nine images of average size around 0.4 ha containing 1,279 trees in total), high-density coniferous forests (seven images of average size around 0.6 ha containing 853 trees in total), and open fields (nonforest) involving trees outside forest in hedgerows and small patches (nine images of average size around 3.4 ha containing 547 trees in total). The F1-score (or dice coefficient) was 0.77, with a recall of 0.69 and a precision of 0.96, indicating that the model underpredicted tree crowns, but most of the predictions were indeed trees. The F1-score was slightly higher for deciduous trees (0.80), while lower for coniferous trees (0.76) and nonforest trees (0.74). The tree counting performance was relatively high, with a coefficient of determination (or *R*^2^) score of 0.93 [see Eq. (6) in the Methods section], a relative mean absolute error (rMAE) of 16.0% [mean absolute error (MAE) = 35 trees/ha], and a relative bias of 10.3%. For deciduous trees, the *R*^2^ score was 0.88, the rMAE was 18.1% (MAE = 61 trees/ha), and the relative bias was 6.1%. For coniferous trees, the *R*^2^ score was 0.88, the rMAE was 13.1% (MAE = 42 trees/ha), and the relative bias was 3.1%. While for nonforest trees, the performance dropped, with a *R*^2^ score of 0.83, a rMAE of 18.9% (MAE = 3.3 trees/ha), and a relative bias of 20.2%. We also fitted a linear regression between the predictions and the references to illustrate how our predictions matched with the references in tendency (Fig. 3B for tree counts and Fig. 3C for crown areas). We noticed an overall high agreement of tree counts as reflected by a close to unity slope of 0.97. However, we noticed an overcount tendency in dense coniferous forests (slope = 1.07), yet an undercount tendency in dense deciduous forests (slope = 0.91) and open areas (slope = 0.88) (Fig. 3B). We also noted that the model underestimated the crown area by ∼20% (Fig. 3C) regardless of the tree density or types, with a relative bias of −22.6% (−22.1% for deciduous trees, −22.4% for coniferous, and −24.1% for nonforest trees), which was likely due to the special attention given on the crown gaps for improving the separability of individual trees. Note that a negative bias indicates underestimation by definition [Eq. (8)].

### Individual tree height prediction from aerial images

The height prediction model received multiband aerial images as inputs and learned the mapping of the reference height obtained from the LiDAR data through a similar U-Net architecture as used in the previous section. To account for the height differences among various landscape types, we constructed a training data set by sampling aerial images from regions dominated by deciduous, coniferous, and nonforest trees with a ratio of 18:18:1 (66) (Fig. S3a). The data set contained in total 74 images (7,400 ha) captured in 2018, with the corresponding LiDAR height data collected primarily from 2018 and partially from 2019 (due to lack of coniferous trees). The pixel-level height prediction, combined with the individual tree crown segmentation, yielded the individual tree height, which we defined as the 95th percentile height within each predicted tree crown.

**Fig. 4.**
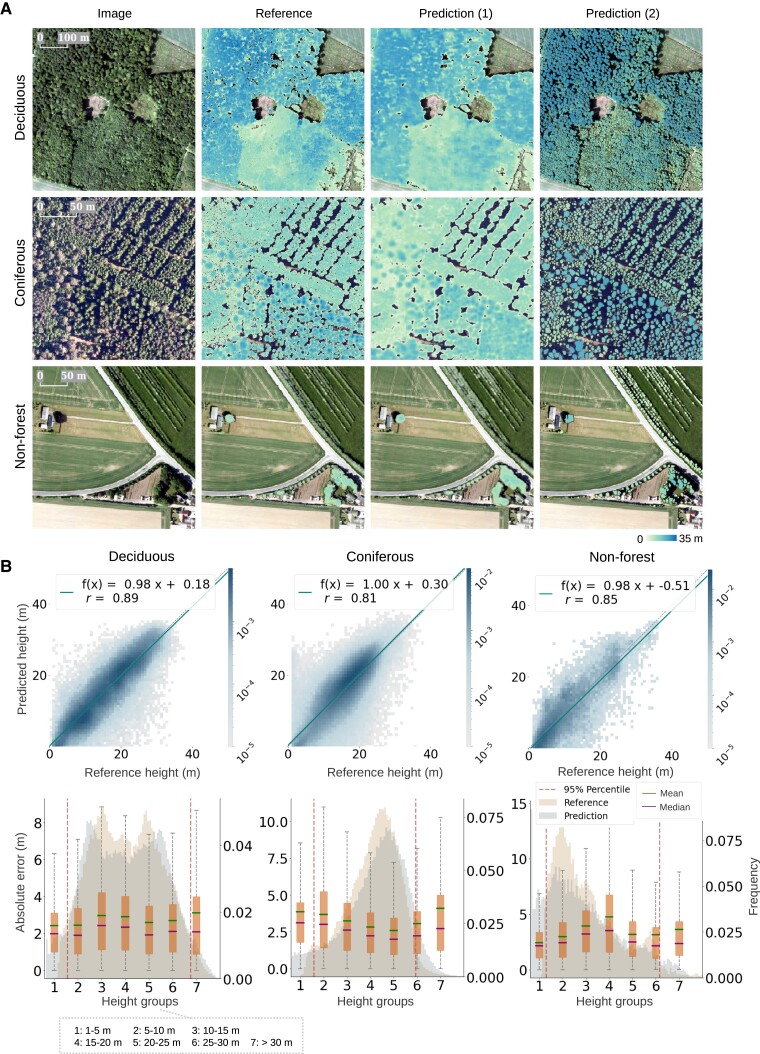
Individual tree height prediction from aerial images. The evaluation was done on the standalone testing data, which contained regions randomly sampled from approximately one-third of Denmark (Fig. S3b). A) Height predictions for three different forest/landscape types: deciduous, coniferous, and nonforest. Prediction (1) shows the height prediction per pixel, and prediction (2) shows the height prediction per tree obtained by using the 95th percentile value from prediction (1) within each segmented tree crown. B) Comparison between predicted individual tree heights, i.e. prediction (2) and reference tree heights derived from LiDAR, with the regression lines shown in solid and the identity lines shown in dotted. Absolute errors of the individual tree height prediction [prediction (2)], grouped in 5-m height intervals, with the predicted and reference height distributions in the background.

We conducted large-scale individual tree height evaluation on the standalone testing data, which contained randomly sampled aerial images (in total 3,000 ha containing 478,328 predicted tree crowns) captured in 2018 and 2019 (in total 1.8 million ha, approximately one-third of Denmark, Fig. S3b), respectively, for each forest/landscape type (Fig. 4). The model achieved a global MAE of 2.9 m [median absolute error = 2.3 m, rMAE = 19.3%, root mean squared error (RMSE) = 3.9 m, and relative RMSE (rRMSE) = 25.9%]. The MAE for deciduous trees was 2.7 m (rMAE = 17.7%, RMSE = 3.6 m, and rRMSE = 23.8%), with a relative bias of −1.3%. However, we observed lower performance for coniferous and nonforest trees with rMAE of 19.5% (MAE = 3.0 m and bias = 2.5%) and 31.1% (MAE = 3.2 m and bias = −15.4%), respectively. The relative bias was relatively high (−3.7% and −8.1%) for short (1–10 m, 23.0% of all trees) and tall (>30 m, 0.7% of all trees) trees, but rather low (1.3%) for medium (10–30 m, 76.4% of all trees) trees. We also fitted a linear regression between the predictions and the references to illustrate how they match in tendency and noticed a reasonable agreement across all forest/landscape types (Fig. 4B). When decomposing the mean squared errors (MSEs) into squared bias and variance (67), we found the biases were generally low except for the tall tree groups (>30 m), while the variation remained high across diverse height ranges and forest/landscape types (Fig. S4a), which agreed with the wide scattering in Fig. 4B.

### Field evaluation and nationwide implementation in Denmark

**Fig. 5.**
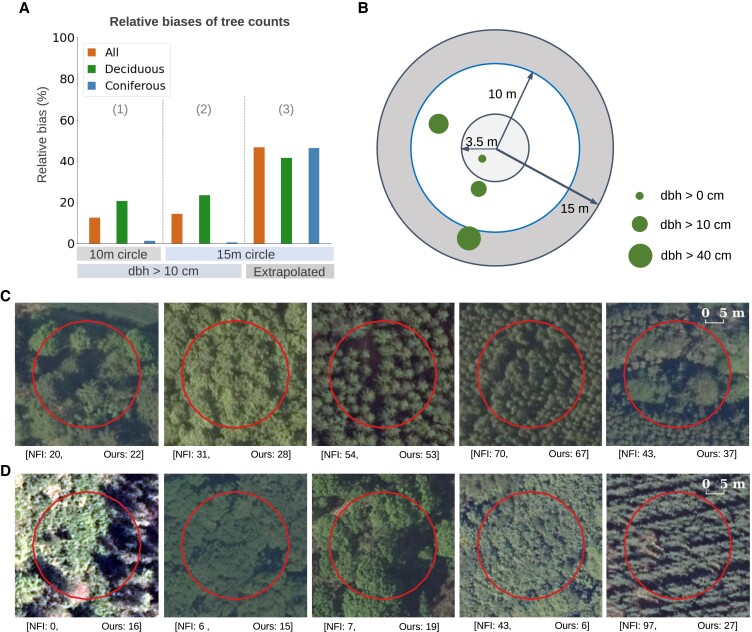
Plot-level evaluation of tree count prediction against NFI field plots. A) Relative biases of tree counts were evaluated in three ways by comparing predicted tree counts against the following: (1) all trees (dbh > 10 cm) in the 10-m radius plots; (2) all trees (dbh > 10 cm) in the 15-m radius plots; and (3) all trees taller than 1.3 m (trees below this minimum height were not measured by the NFI), where the numbers of smaller trees not measured exhaustively on the field were extrapolated by the NFI. Biases were calculated for all plots, plots with deciduous trees, and plots with coniferous trees, separately. Absolute bias values are displayed for clear visualization. B) Design of the Danish NFI. Each field plot consists of three concentric circles with a radius of 3.5, 10, and 15 m. Trees taller than 1.3 m are measured exhaustively in the inner circle (3.5-m radius), trees with dbh > 10 cm are measured exhaustively in the middle circle (10-m radius), and trees with dbh > 40 cm are measured exhaustively in the entire circle (15-m radius). The NFI extrapolates trees of all sizes to all circles, assuming a homogeneous distribution. C) Examples of tree counts from NFI and our predictions with low bias. Here, the NFI counts refer to the number of trees (dbh > 10 cm) in the 15-m circle. D) Examples of tree counts from NFI and our predictions with high bias.

We conducted an independent plot-level evaluation for tree counts against 2,563 field plots involving 18,588 tree records from the Danish NFI in 2018 (68) (Fig. 5; see plot locations in Fig. S5). Each field plot consisted of three concentric circles (Fig. 5B), where small trees [diameter at breast height (dbh) < 10 cm] were only measured in the inner circle (3.5-m radius), trees with dbh > 10 cm were only measured in the middle circle (10-m radius), and trees with dbh > 40 cm were measured in the entire plot (15-m radius) (see also the Methods section) (68). In the 10-m radius circles, the relative bias against inclusive counts for all trees measured larger than 10 cm in dbh was 12.5% (20.6% for deciduous plots and 1.3% for coniferous plots). When extrapolating trees with dbh > 10 cm to the 15-m radius circles (assuming an even distribution of stems), the bias was 14.3% (23.4% for deciduous plots and 0.5% for coniferous plots). Smaller trees were not systematically counted on the field within these plots (15-m radius), so the numbers above quantified the errors for larger trees (dbh > 10 cm). To evaluate our underestimation of small or understory trees (the NFI height limit for trees is 1.3 m), we extrapolated the small trees counted in the inner circles to the entire plot. Here, the bias was −46.6% (a negative bias implies underestimation). We also observed that the bias was relatively low (−17.6%) for plots where large trees (dbh > 10 cm) comprised more than half of the total extrapolated number of trees, while much higher (−80.3%) for plots dominated by small trees (dbh < 10 cm), implying an expectedly large underprediction of small trees, which are outshaded by tall trees. The uncertainties inherent in the extrapolation approach for small trees could also be considerable (see Fig. S6).

The plot-level comparison against the field data across various landscape types reflected robustness as well as limitations of our framework in different environmental settings. The biases tended to be low for trees with rather clear crown structures, regardless of stand density or species (Fig. 5C). Meanwhile, we noticed severe biases for meadows with tree-like structures, trees with ambiguous or multiple branches, or thin and tall coniferous trees with highly inclined shadows. For exceedingly dense forests with no evident crown gaps, both overprediction and underprediction might occur (Fig. 5D).

We evaluated the scalability of the framework by generating an individual tree count map and a crown segmentation map featured with individual tree heights for Denmark (Figs. 2 and S7). A total of 312 million trees were detected and a total crown area of 0.47 million ha was predicted (Table 1). The results revealed a surprisingly large number of nonforest (66) trees (91 million), which represents around 30% of the national tree crown coverage. Compared with the Danish NFI forest tree count from 2018 (69), which upscaled field measured plot information to nationwide forest areas, our predictions showed an underestimation bias of 77.6%. Note however that 50.9% of all trees from the NFI estimates were <4 m in height and that the number extrapolated from plot level to national scale may include various sources of uncertainty.

**Table 1.** Tree count, total crown area, and tree height products for Denmark, grouped in three major forest/landscape types.

Forest/landscape type	Tree count	Tree crown area (ha)	Tree height [mean (50%, 95%), m]
Deciduous forest	136,467,592 (43.7%)	233,720 (50.0%)	12.4, [11.6, 25.5]
Coniferous forest	85.023,211 (29.1%)	92,352 (19.8%)	13.2, [12.8, 24.4]
Nonforest	91,014,130 (27.2%)	141,186 (30.2%)	6.6, [5.4, 18.2]
Total	312,504,933 (100%)	467,257 (100%)	11.1, [10.5, 24.2]

Additionally, we compared the tree cover map aggregated from our individual tree crown segmentation with two existing state-of-the-art forest cover maps estimated from satellite imagery at 30- (Landsat) (11) and 10-m (Sentinel-2) (66) resolutions and noticed a much higher tree canopy area in dense forests from these existing products (Fig. S8a). In particular, the 10-m resolution Copernicus tree cover map (2018) (66) showed 32.9% higher values for deciduous forests and 50.7% for coniferous forests and conversely 50.3% lower for nonforest areas (Fig. S8b and Table S2). We believe that such discrepancy was potentially induced by the differences in the detectability of trees from different resolutions and the interpretation of crown gaps or shadows.

### Transfer learning enables cross-national applications

**Fig. 6.**
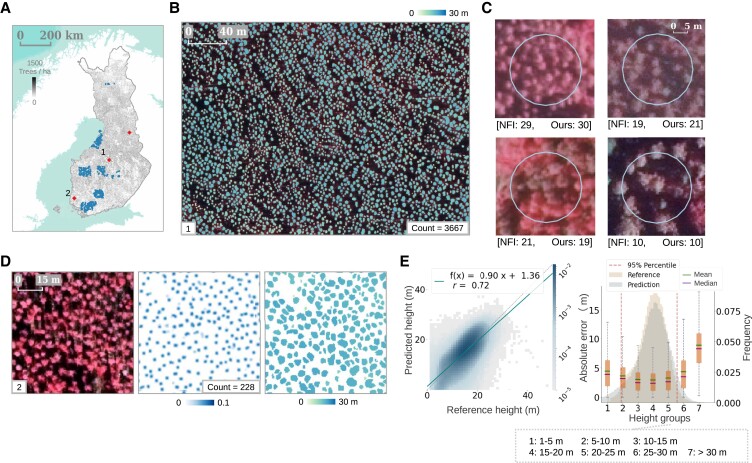
Transferring the proposed framework from Denmark to Finland. A) Tree count prediction in Finland derived from aerial images using the fine-tuned model. Locations of the evaluations in B), D), and E) are shown in diamond shapes. Locations of the tree count evaluation against NFI field data are shown in dots. B) Large-scale examples of individual tree crown segmentation colored by height prediction. C) Examples of the predicted tree counts evaluated against the NFI field plots (12.62-m radius). The NFI tree counts represent the number of trees taller than 1.3 m measured in each plot on the field. D) Detailed examples for individual tree counting, crown segmentation, and height prediction. E) Comparison of predicted tree heights and reference tree heights, with the regression line in solid and the identity line in dotted. Absolute errors for the evaluation of individual tree height prediction.

The proposed framework was tested for transferability regardless of the input data source, spatial resolution, composition of the spectral bands, and differences in major forest/landscape types. The models pretrained with the Danish data set at 20-cm resolution were easily adapted to 50-cm aerial images from Finland by fine-tuning using a small additional training set of the target distribution (Fig. 6). Specifically, the counting and crown segmentation model established for Denmark was further trained (or fine-tuned) with the original data from Denmark and additional data from Finland including up-sampled coarser resolution images and 4,773 tree crown delineations (Fig. S9a). Likewise, the pretrained height prediction model using data from Denmark was adapted to the Finnish setting by fine-tuning with 10,800 ha of images and 1-m resolution LiDAR height data collected from three locations in Finland (Fig. S9b).

We evaluated the tree counting performance using 1,645 Finnish NFI field plots (12.62-m radius) collected in 2019 (70) (see plot locations in Fig. 6A). For each field plot, the tree count predictions from aerial images captured in 2019 were compared against the number of tree trunks from the inventory data (see examples in Figs. 6C and S10). The relative bias was −26.2% for all plots. The bias was low (−8.0%) for less dense (tree count < 500/ha) plots (43.5% of all plots), yet higher (−40.2%) for highly dense (tree count > 500/ha) plots (56.5% of all plots). Notably, the NFI data included all trees taller than 1.3 m, and trees branched below breast height were counted separately for each branch (70). We evaluated the performance of individual tree height prediction on randomly sampled images (3,000 ha containing 925,597 predicted tree crowns) from three regions in Finland (21,600 ha), where images and LiDAR height data were collected during the same period (2019). The selected regions were dominated by either coniferous forests or nonforest areas (66). The predicted tree heights showed a reasonable agreement with the reference heights (Fig. 6e), and the MAE was 3.1 m (rMAE = 21.5%, median absolute error = 2.6 m, RMSE = 4.1 m, rRMSE = 28.0%, and relative bias = −0.4%; see also Fig. S11). The absolute errors increased for shorter (<5 m) and taller trees (>30 m) (Fig. 6E).

Notably, when the models trained for Denmark were directly applied to Finland, reduced performances were observed (see Fig. S12). The Finnish forests are mostly managed, and to do further tests on unmanaged forests, we also applied the models trained with the Danish data on data sets from other countries with diverse landscape types including mountainous areas. This involved 20-cm resolution RGB aerial images captured in 2018 from France (Baronnies Provençales Regional Nature Park, Fig. S13) and 60-cm resolution RGB + NIR aerial images captured in 2018 from the United States of America (Sierra National Forest, Fig. S14). We noticed that classification models, i.e. the crown segmentation models that involved classification at pixel level, showed higher robustness by capturing most visible tree crowns correctly. In contrast, regression models, such as the height prediction model, were more vulnerable to distribution changes of the input pixel values (Fig. S15) and were unlikely to capture the expected height distribution (Fig. S16). However, such distribution shift could be rectified by fine-tuning using a small data set of local canopy height maps (Figs. S12 and S16).

## Discussion

We established a deep learning-based framework for individual overstory tree mapping and height prediction in forest and nonforest areas from high-resolution aerial images and applied it to two European countries with dissimilar data sets and landscape types. Our approach enabled the derivation of the height information, normally only available from high-cost LiDAR data, from less expensive aerial imagery (62, 63, 71). While aerial images cannot be considered “low cost,” the availability of submeter resolution images from nanosatellites, such as SkySat (72), provides a comparable quality for a reasonable price (73, 74). We propose such an individual tree localization and characterization approach as the means to produce a comprehensive tree database with a focus on nonforest trees, which are essential yet often not systematically investigated by conventional forest inventories (9). The quantification of these nonforest tree resources is important, as we found that following existing forest maps and definitions, about 91 million trees in Denmark were located outside forests and thus were not part of the NFI statistics (69). Taking advantages of the high-resolution aerial data, our method unambiguously determined trees as separate objects. Such information could supplement the existing coarser resolution (10–30 m) tree cover products derived from coarser spectral data (11, 66). Particularly, the comparison against the Copernicus map suggested over 50% underestimation of tree cover in nonforest regions. Moreover, carbon stocks could be reported for individual trees outside forests, for example in urban areas. Using local or global allometric equations (53), crown diameter and height of the detected trees could be directly converted to carbon stocks (75). The localization of individual trees is also particularly important for the monitoring of mortality of large trees (dbh > 10 cm), which would not be based on plot-scale estimations, but on actual counts with wall-to-wall coverage (76, 77).

Nevertheless, individual tree counting and crown segmentation were subject to several uncertainties and limitations. Firstly, the manual delineations of tree crown references are a source of uncertainty. We excluded small trees, shrubs, and bushes with no visible shadow or with a crown area below 0.08 m^2^. Yet, labeling individual trees was not always obvious, particularly for touching or overlying crowns, heavily shadowed crowns induced by nonvertical shooting angles, and the coexistence of single and multibranched trees. Besides, the individual variation in manual labeling (from two independent data labelers) might also aggravate the model performance (78). Despite the uncertainties immanent in the labels, the relatively high performance when evaluating against the manual delineations was encouraging, as it implied that the model could transfer the knowledge it learned from the labels to unseen cases. Secondly, the nature of the aerial image data limited the detection of trees merely to the topmost layer for dense or closed canopies. While larger trees that towered above the surrounding understory could be mapped with a rather low bias, small understory trees (down to a height of 1.3 m), which made up a considerable part of the total tree count, could not be detected. The NFI field data, which include records of small trees, could be used to correct for this bias by adding extrapolated numbers for the understory trees. Lastly, we designed the model to solve a semantic segmentation rather than an instance segmentation problem, as the latter usually involves a more complicated two-step scheme, i.e. detection of bounding boxes followed by regression within the detected boxes (79–81). However, instance segmentation could potentially lead to better separation of adjoining objects (82, 83), i.e. densely connected tree crowns, which may be tested in future studies.

The aerial image-based tree height prediction is particularly challenging since the 2D spectral features are insufficient to fully reflect the third spatial dimension of heights (61). We noticed relatively high errors for the taller trees, which is likely due to the fact that tall trees, despite methodological data balancing, are generally rare and are extremely hard to infer from optical imagery. Particularly, for dense forests where shadows or tree edges were hardly visible, the model tended to be conservative and thus underpredict tree heights. Secondly, the mismatch between the aerial imagery and the LiDAR height data set (Fig. S17) could cause problems for high-resolution studies. The mismatch occurred since regular orthoimages are generated using a terrain model. True orthoimages could be a potential solution for fixing this issue (84). Thirdly, a single model generalized on all trees might not be effective enough to capture the height differences between different tree species with diverse traits. Lastly, the individual tree height was defined as the 95th percentile height within each predicted tree crown, thereby making the tree height products dependent on the individual tree crown segmentations. If the segmentation model had a systematic bias, then it might be propagated to the error calculation of the height model. Such bias could accumulate for short trees that tend to have small crowns (Fig. S4b).

Uncertainties could also arise when transferring the established models to markedly different regions, considering the divergences in forest type distributions, traits of trees affected by local climate, image acquisition times, viewing angles, and spatial resolutions. Yet, our study revealed the feasibility of pretrained models for automatic individual tree localization and characterization in different data sets featuring diverse landscape types, which could be further extended with the availability of high-quality aerial or satellite imagery.

Here, we publish a readily available framework pretrained on data samples from Denmark and Finland, which can be adapted to other domains by fine-tuning with a little extra data from specific landscape types. This may enable countries to make use of their aerial images to derive annual airborne inventories of overstory trees with a manageable effort (see examples for France and the United States of America in the Results section). A database on individual overstory trees could allow more sophisticated and attentive utilization of wood material, as wood properties are influenced by the local growing conditions, leading toward resource efficiency and sustainable utilization of trees. Our proposed framework may thus support the monitoring of tree resources toward a digitalized environmental management in support of the green transition.

## Methods

### Tree counting and crown segmentation

We used a multitask deep neural network with two partially connected branches for the tree counting and crown segmentation tasks. The crown segmentation branch solved a semantic segmentation problem, where each pixel in a given image was classified as either object or background (85). The second branch predicted the tree count by regressing density maps for a given image. The ground truth density maps were generated by applying normalized 2D Gaussian kernels on the manual crown delineations (60, 86). Following the strategy from Zhang et al. (86), given an image with a total of *C* tree delineations, the density map *D* is defined as in Eqs. (1) and (2):


(1) $$G_{\sigma },m(p)=\{\begin{array}{cc} e^{-\frac{||p-m|{|}^{2}}{2\sigma^{2}}}\cdot {\left(\sum_{r,s=-M}^{M}e^{-\frac{r^{2}+s^{2}}{2\sigma^{2}}}\right)}^{-1} & \mathrm{if}\;m_{d}-M\leq p_{d}\leq m_{d}+M\;\mathrm{for}\;d=1,2\\ 0 & \mathrm{otherwise} \end{array}$$

(2) $$D(p)=\sum_{c=1}^{C}G_{\sigma ,m_{c}}(p).$$

Here, $G_{\sigma ,m}(p)$ is a sampled truncated Gaussian kernel evaluated at pixel position $p=(\;p_{1},p_{2}{)}^{T}$ in the image. The kernel is centered around $m=(m_{1},m_{2}{)}^{T}$ with bandwidth parameter σ and is truncated to height/width 2M+1. D(p) denotes the density map for *C* tree delineations centered at the position $m_{1},\ldots ,m_{C}$ evaluated at pixel position *p*. We used a fixed Gaussian filter with a kernel size of 15×15 [i.e. M=7 in Eq. (1)] and a SD (σ) of 4 (Table S4 and Fig. S19). Through the normalization term in Eq. (1), we ensured that each Gaussian kernel was normalized to unity. The total tree count could then be estimated by summing up the density values across the whole image. Compared with counting by enumerating the segmented tree crowns, where several adjoining tree crowns might be incorrectly counted as one, the density estimation-based approach improved the overall counting bias by 8.9% (Table S5 and Fig. S20).

The network was primarily based on the U-Net architecture (64), a widely used neural network for computer vision applications. Following the approach from Oktay et al. (65), we extended the standard U-Net with self-attention blocks to extract more relevant information from the down-sampling path (details of the model can be found in Table S6). Batch normalization was applied after each convolutional layer to stabilize and to speed up the training process (87). The majority of the model weights were shared across the two branches, while only those for producing the final output predictions were task specific. For the segmentation branch, the sigmoid activation was used in the final output layer to produce probabilities in the range of [0,1], which were then converted into binary labels with a threshold of 0.5. For the counting branch, the linear activation was used to maintain the Gaussian kernel values. In each epoch of training, random patches of size 256×256 pixels from all available training images were extracted to generate training and validation data with a batch size of 8. The generated image patches were standardized (per instance and per channel) to 0 mean and unit SD before being fed into the network as inputs. We used the Adam optimizer (88) for training.

We manually delineated a total of 24,466 individual tree crowns from sampling plots of varying sizes distributed over Denmark, among which 19,771 crowns (49% in dense deciduous forests, 30% in dense coniferous forests, and 21% in nonforest areas) were assigned to the training data set, 2,016 crowns (46% in dense deciduous forests, 38% in dense coniferous forests, and 15% in nonforest areas) to the validation data set, and 2,679 crowns (48% in dense deciduous forests, 32% in dense coniferous forests, and 20% in nonforest areas) to the final test data set. The manual delineation took ∼3 weeks and involved creating annotations covering a large variety of tree species and landscape types. No semiautomatic assistance was applied, to ensure that all labeled tree crowns consistently represented what a human eye could see from an aerial image. Practically, delineating individual deciduous tree crowns in dense forests was more difficult than delineating coniferous tree crowns. We observed that some deciduous trees have complex canopies forming clumps, and thus, the crown boundaries are not always clear from an aerial image point of view, while coniferous tree crowns are more separable due to their cone-shaped appearance. The network was trained in a fully supervised manner with the training data set. The validation data set was used for model selection and hyperparameter tuning (including the Gaussian parameters and the gap penalty weight). The test data set was only used for the final evaluation (see the Results section and also Table S9). For training the two branches, we generated two types of target outputs from the referential annotations: (i) binary masks with tree pixels denoted as ones and background pixels as zeros for the segmentation branch and (ii) density maps with single trees represented by Gaussian kernels for the counting branch.

The network, when randomly initialized, could be retrained from scratch using any composition of input bands, resulting in several final models with slightly different performances (Table S1 and Fig. S1). The architecture of the network could also be modified slightly to allow for multiresolution input images (details of the model can be found in Table S7). Specifically, the bands with the highest spatial resolution would be fed into the topmost input layer, while the bands with coarser resolutions would be fed into the network after specific down-sampling layers when the spatial resolutions matched. In our experiments, the input data consisted of aerial images (RGB + NIR bands) with a spatial resolution of 20 cm and canopy height maps at 40-cm resolution derived from LiDAR data. The coarser resolution height maps were fed into the network after the first down-sampling layer.

The model was trained by minimizing a combined loss $l_{\mathrm{seg}\_\mathrm{count}}$ from the two branches. The segmentation loss $l_{\mathrm{seg}}$ was based on the Tversky index (89), a generalized version of the F1-score (90), which penalizes false positives and false negatives differently. To account for the segmentation failures in separating densely connected or overlaid tree crowns, we highlighted the between-crown gaps. Specifically, crown gap maps were generated based on the crown delineations by morphological operations, with the gap pixels being assigned a higher weight than other pixels. The pixel-wise weights were applied in the loss computation so that the misclassified gap pixels were penalized more heavily than others (50, 64). Given a training image $I_{n}$, let $p_{0i}\in [0,1]$ denote the predicted probability of pixel $i\in I_{n}$ being an object. Let $g_{0i}=1$ if *i* is an object and $\;g_{0i}=0\;$ otherwise, and let $p_{1i}=1\;-\;p_{0i}$ and $g_{1i}=1-g_{0i}$. Suppose $w_{i}$ indicates the weight for pixel *i*, the pixel-wise weighted Tversky loss was then defined as shown in Eq. (3) (91):


(3) $$l_{\mathrm{seg}}=1-\frac{\sum_{i\in I_{n}}w_{i}p_{0i}g_{0i}}{\sum_{i\in I_{n}}w_{i}p_{0i}g_{0i}+\alpha \sum_{i\in I_{n}}w_{i}p_{0i}g_{1i}+\beta \sum_{i\in I_{n}}w_{i}p_{1i}g_{0i}}.$$

We adjusted the penalty weight assigned to the between-crown gaps to optimize both the mapping of crown coverage and separability of the crowns. With an increasing gap penalty from low (1) to high (10), individual crowns could be separated more clearly (counting bias reduced from −16.5 to −7.9%), while the predicted crown area declined (relative bias increased from −16.6 to 27.5%, Table S3 and Fig. S19). We found a gap penalty of 5 to adequately balance both the individual tree separation and crown area accuracy (Fig. S18).

For the counting branch, the pixel-wise MSE, as defined in Eq. (4), was employed as the loss function for evaluating the differences between the predicted density map $D^{\mathrm{pred}}$ and the ground truth density map $D^{\mathrm{gt}}$:


(4) $$l_{\mathrm{count}}=\frac{1}{M}\sum_{i=1}^{M}{\left(D^{\mathrm{pred}}(i)-D^{\mathrm{gt}}(i)\right)}^{2}.$$

Here, *M* denotes the number of pixels in the image. The total loss was a weighted summation of the segmentation loss and the density estimation loss: $l_{\mathrm{seg}\_\mathrm{count}}=l_{\mathrm{seg}}+\lambda_{t}l_{\mathrm{count}}$. The weighting factor $\lambda_{t}$ was initially set to 100 and increased steadily during training to ensure that the two losses were rescaled to a similar magnitude. The final model was determined as the one achieving the lowest error on 800 randomly chosen validation patches. We trained the model for 1,500 epochs (32 h on one GPU), and the training and validation curves are shown in Fig. S21.

### Individual tree height prediction

#### Canopy height prediction

We formulated the canopy height prediction task as a pixel-wise regression problem. Given a set of multiband aerial images $\left\{I_{1},I_{2},\ldots ,I_{n}\right\}$ (20-cm resolution) and a set of corresponding LiDAR-derived canopy height maps $\left\{H_{1},H_{2},\ldots ,H_{n}\right\}$ (40-cm resolution), a pixel-wise mapping from *I* to *H* was established. To balance the differences in different height groups and forest type distributions, we randomly sampled 45 aerial images (4,500 ha) from regions dominated by coniferous forest (collected in summer 2018 and 2019) and deciduous forest (66) (collected in summer 2018) with an average LiDAR height over 8 m, respectively. Besides, three aerial images covering 300 ha were randomly sampled from all available nonforest (66) area images taken in summer 2018. The whole data set was split into a training (74 images) and validation (19 images) set with a ratio of 4:1 using stratified sampling. The training data were used for learning the model parameters, and the validation data were used for model selection. The final evaluation was done using a standalone test data set (see the Results section). Note that there was no spatial overlap between the three data sets (training, validation, and testing) (see also Table S9). The input multiband aerial images were globally standardized to 0 mean and unit SD based on the training data set before being fed into the network. Data augmentation techniques (92) including random flipping, cropping, Gaussian blurring, and brightness adjustment were applied during training. The network shared a similar U-Net architecture (64, 65) as the counting branch of the multitask network, with the last activation function being a linear transformation (details of the model can be found in Table S8). The final decoding block was removed due to the coarser resolution of the LiDAR height maps. A weighted MAE (wMAE) was used as the loss function, where heights over 10 m were given a higher weight *w* of 5 to penalize particularly the underprediction of the taller trees. Denoting the reference and the predicted tree height at pixel i∈I as $y_{i}$ and ${\hat{y}}_{i}$, the pixel error $l^{I}(i)$ was formulated as Eq. (5):


(5) $$l^{I}(i)=\{\begin{array}{cc} \left|y_{i}-{\hat{y}}_{i}\right| & y_{i}<10\;m\\ w*|y_{i}-{\hat{y}}_{i}| & y_{i}\geq 10\;m \end{array}.$$

We trained the model for 1,000 epochs (20 h on one GPU), and the training and validation curves are shown in Fig. S22. After training, we further adjusted the parameters in the last layer to minimize the systematic errors normally caused by iterative minibatch neural network learning in practice (93). The adjusting coefficients were obtained using the validation data. The unadjusted predictions were fitted against the reference heights using a linear regression (Fig. S23). Consider a fitting equation in the form y=ax+b, where *y* indicates the original prediction, *x* indicates the reference, and *a* and *b*indicate the coefficients. The adjusted prediction can be calculated by $y^{\prime }=\;\frac{y-b}{a}$. The resulting coefficients from the fitting were then applied to adjust the original predictions. When tested on the independent testing data, we observed that the adjustment improved the overall bias [Eq. (9)] from 13.5 to 1.3% for deciduous trees, from 13.2 to 2.3% for coniferous trees, and from 14.8 to 7.4% for nonforest trees.

#### Individual tree height prediction

Individual tree heights were obtained by combining the canopy heights with the individual tree crown segmentation results through the following steps. First, the predicted individual tree crowns were polished by removing tiny segments with an area of <2 pixels (0.08 m^2^ on site). Second, to mitigate the uncertainties induced by slight mismatches between the aerial images and LiDAR data (84) (Fig. S17), each predicted tree crown was expanded by a distance ($d=\alpha \sqrt{s/\pi }$) proportional to its area *s*. We set the expanding factor α to 0.2 in the experiments. Finally, the 95th percentile height within each refined tree crown was determined as the tree height. The individual tree-level height prediction performance was evaluated by comparing the referential and the predicted tree height derived from the LiDAR height references and the canopy height predictions. Notably, suspicious tree height references (<0.1% of the whole testing set) were removed by filtering out tree crowns with a maximal NIR value higher than 80 yet a maximal height lower than 1 m, as a high NIR value is normally closely associated with vegetation and is therefore regarded as an anomaly when the height is low. We determined the threshold value of 80 experimentally by testing different values and found that it could properly differentiate vegetation from nonvegetation and was able to help remove trees with doubtful reference heights (Fig. S24). Detected trees with reference heights lower than 1 m were removed (<0.2% of all trees), as they were too low to be considered trees according to common NFI definitions (1.3 m) (68, 70).

### Transfer learning and cross-national applications

The individual tree counting and crown segmentation model was adapted to Finland by fine-tuning the pretrained weights established on the data from Denmark (using G + B + NIR bands as inputs). The fine-tuning data set consisted of the original data set from Denmark (19,771 tree crown delineations in 84 plots) and a small data set from Finland (4,773 tree crown delineations in 19 plots), which was oversampled five times to balance the two data sets. The aerial images at 50-cm resolution in Finland were up-sampled to 25 cm using bilinear interpolation to match with the 20-cm images from Denmark.

The height prediction model was adapted to Finland by fine-tuning with a canopy height data set from Finland. The Finland data set consisted of three aerial images (10,800 ha, G + B + NIR bands) at 50-cm resolution and the corresponding LiDAR height data at 1-m resolution, both collected in 2019. The multiband aerial images were up-sampled to 25-cm resolution and globally standardized to 0 mean and unit SD before being fed into the model. The LiDAR height data were up-sampled to 50-cm resolution accordingly. The wMAE loss [Eq. (5)] was also used for fine-tuning the model.

Additionally, we tested the models trained using only the Danish data on data sets from other countries without any transfer learning. This includes Finland (GB + NIR aerial images at 50-cm resolution), France (RGB aerial images at 20-cm resolution), and the United States of America (RGB + NIR aerial images at 60-cm resolution). For aerial images with the same resolution (French data), we applied the Danish model directly without any adjustments of configurations. For images with lower resolution (Finish and the United States of America data), we up-sampled the images by a factor of 2 before applying the Danish model.

### Evaluation against NFI field plot data

#### For Denmark

We evaluated our tree count predictions with the Danish NFI field plot data collected in the same year as the aerial images (2018). Each field plot consisted of three concentric circles with radius of 3.5, 10, and 15 m. All trees taller than 1.3 m were measured exhaustively in the inner circle (3.5-m radius), trees with dbh larger than 10 cm were measured exhaustively in the middle circle (10-m radius), and trees with dbh larger than 40 cm were measured exhaustively in the entire circle (15-m radius) (see Fig. 5B). Exact locations of the measured trees were unavailable, and the tree counts had been summarized in a way that exhaustive count of trees larger than 10 cm in dbh could not be obtained for a certain area. Note that the Danish NFI scheme was delicately designed for efficient field-based studies other than for evaluations such as used for our study, and therefore, a certain discrepancy cannot be excluded. Conventionally, the total number of trees could be estimated by extrapolating the measured numbers based on the ratio of areas, assuming an even distribution of stems (68). For instance, for an area of size $A_{1}$ with *N* measured trees, the number of trees in an area of size $A_{2}$ can be calculated by $N\times \frac{A_{2}}{A_{1}}$. Similarly, we estimated the total number of trees with dbh larger than 10 cm in the 15-m circle by extrapolating trees not measured outside of the 10-m circle (dbh < 40 cm) according to the ratio of areas (2.25). We also estimated the total number of trees (dbh > 10 cm) in the 10-m circle by removing trees measured outside of the 10-m circle (dbh > 40 cm) according to the ratio of areas (2.25). We noticed that these two approaches induced comparable bias results (Fig. 5a), indicating that the extrapolation was reasonable. Lastly, we compared our predictions with the estimated total number of trees (dbh > 0 and height > 1.3 m) in the entire plot (15-m radius), the latter calculated by NFI with additional factors involved in the extrapolation approach (e.g. forest fraction) (68).

#### For Finland

We evaluated our tree count predictions with the Finish NFI data collected in the same year as the aerial images (2019). We used the field plots for grown-up trees (12.62-m radius circles), thus excluding the nursery experiment study areas. In the Finnish NFI design, trees are defined as perennial tree-stemmed plants measured taller than 1.3 m in height with a distinct trunk and are exhaustively counted for each plot (70).

### Production of countrywide maps and comparison with existing forest cover products

#### For Denmark

We applied the established tree counting and crown segmentation model as well as the height prediction model for Denmark. The final nationwide products include an individual tree crown segmentation map (20-cm resolution), a tree count density map (20-cm resolution), and a canopy height map (40-cm resolution; see examples in Fig. S7).

The predicted individual tree crowns consistently underestimated the true annotated crown area by ∼20% (Fig. 3C). Such underestimation was inherent in the design of the study as crown gaps were intentionally highlighted to ensure the separation of densely connected tree crowns. The errors would accumulate for tree cover estimation at scale and likely lead to severe underestimation of total crown cover estimation. To obtain an unbiased total crown area at a country level, we corrected for the underestimation by upscaling the predicted crown area according to the linear relationship obtained by fitting the predicted crown area (as *y*-axis) against the reference crown area (as *x*-axis) (Fig. S25) (93). Consider a fitting line in the form y=ax+b, where *x* indicates the reference and *y* indicates the prediction. The corrected prediction can be calculated by $y^{\prime }=\;\frac{y-b}{a}$. The correction parameters were obtained using the validation data. When tested on the standalone testing data (described in the Results section), the correction improved the overall bias [Eq. (9)] of crown area from 23.4 to 0.1% for deciduous trees, from 25.4 to 0.1% for coniferous trees, and from 28.1 to 0.3% for nonforest trees (see Fig. S25). The low overall biases indicated that a simple correction step could help mitigate the inherent limitations of the individual tree-based approach and lead to higher performance that could nearly reproduce manual annotations.

We generated tree crown cover (%) maps by aggregating individual crown areas at coarser spatial resolutions (10 and 100 m). Compared with similar existing forest/tree cover products (11, 66), our crown cover maps showed visually lower values (Fig. S8a). We numerically compared our crown areas with the Copernicus tree cover areas from the same year (2018) (66) for each forest type and observed lower estimates of our crown areas in forest regions yet higher estimates in nonforest regions (Fig. S8b). Numerous nonforest trees that were mainly found in hedgerows invisible from low-resolution satellite imagery were clearly visible from high-resolution aerial images. The comparably low estimates of forest cover resulting from our analysis were partially due to the differences in the detectability of trees caused primarily by resolution differences. By inspecting the high-resolution aerial images, we manually annotated the part of tree crowns visible from an aerial image perspective, therefore excluding shadows or between-crown gaps. Notably, tree crowns annotated by inspecting the aerial image data and tree crowns observed in the field would not match perfectly due to viewing angles, branches, observation time, etc. Oppositely, the Copernicus maps used the common forest/tree cover definition, which estimated the percentage of tree cover at a coarser resolution (≥10 m) (Fig. S8c). We evaluated the bias caused by the visibility differences by plotting crown areas obtained from our manual delineations against those from the Copernicus maps. The Copernicus-based crown area was 63% higher than the delineation-based crown area, indicating a systematic bias (Fig. S8d). In conclusion, aggregated individual tree crown area and the existing coarser resolution forest cover maps both reflect the growing condition of trees, but from distinct aspects.

#### For Finland

We applied the tree counting and crown segmentation model for Finland using aerial images (50-cm resolution) captured between 2010 and 2020, since only a fraction of the entire country was scanned yearly. We noticed that combining aerial images from multiple years induced inconsistent results due to removal or restoration of trees and dissimilar image quality or configurations. Further investigation or adjustments may improve the performance.

### Evaluation metrics

For evaluating the segmentation performance against manual delineations, we computed F1-score (also known as the dice coefficient), recall, and precision. For evaluating the counting performance against manual delineations, we computed the coefficient of determination (*R*^2^ score) defined as in Eq. (6), the MAE and the rMAE as defined in Eq. (7), and the relative bias as defined in Eq. (8) (94, 95).


(6) $$R^{2}\;=\;1-\frac{\sum_{i}\;{\left(y_{i}-{\hat{y}}_{i}\right)}^{2}}{\sum_{i}\;{\left(y_{i}-\bar{y}\right)}^{2}}.$$

(7) $$\mathrm{relative}\;\mathrm{MAE}\;=\;\frac{\frac{1}{n}\sum_{i}\;|y_{i}-{\hat{y}}_{i}|}{\bar{y}}.$$

(8) $$\mathrm{relative}\;\mathrm{bias}\;=\;\frac{1}{n}\overset{n}{\underset{i=1}{\sum }}\frac{\left({\hat{y}}_{i}-y_{i}\right)}{y_{i}}.$$

Here, *y*, $\hat{y}$, and $\bar{y}$ denote the reference, prediction, and the mean reference value, respectively, and *n* denotes the total number of samples (tree crowns). Note that the relative bias can also be defined by swapping $\hat{y}$ and *y* in the numerator, which results in the opposite of the value obtained from Eq. (8) (53).

For evaluating our tree count predictions against the NFI field data, we used the relative bias [Eq. (8)] calculated plot by plot.

For evaluating the tree height predictions, we computed MAE, rMAE, median absolute error, RSME, rRMSE, and relative bias. The MSEs were further decomposed into squared bias (the first term) and mean squared variation (the second term) (67), i.e. $l=(\bar{y}-\bar{\hat{y}}{)}^{2}+\frac{1}{n}\overset{n}{\underset{i=1}{\sum }}\;[(y_{i}-\bar{y})-({\hat{y}}_{i}-\bar{\hat{y}}){]}^{2}$.

To quantify the bias for large-scale aggregated results (e.g. aggregated crown area), we computed the overall bias defined in Eq. (9) (93), which is a similar bias metric as the relative bias in Eq. (8), but evaluated at the aggregated level rather than at the sample level.


(9) $$\mathrm{overall}\;\mathrm{bias}=\;\frac{\left|\sum_{i}\;({\hat{y}}_{i}-y_{i})\right|}{\left|\sum_{i}\;y_{i}\right|}.$$

## Supplementary Material

pgad076_Supplementary_Data

## Data Availability

The aerial imagery from spring and the LiDAR-derived canopy height maps are free and publicly available at https://dataforsyningen.dk/. The user rights of aerial imagery from summer (as used for this study) are generally subject to a fee, and the ownership and user rights of these images differ annually. The use of the imagery in research applications may be granted free of charge. Contact the Danish Agency for Data Supply and Infrastructure for more information. The aerial images and canopy height maps from Finland are free and publicly available at https://www.maanmittauslaitos.fi/en/maps-and-spatial-data/. The aerial images from France are free and publicly available at https://geoservices.ign.fr/bdortho. The aerial images from the United States of America are free and publicly available at https://naip-usdaonline.hub.arcgis.com/ (the National Agriculture Imagery Program (NAIP)). The Copernicus forest cover maps and forest type maps (2018) for Denmark and Finland were downloaded from https://land.copernicus.eu/pan-european/high-resolution-layers/forests. We publish our models at https://github.com/sizhuoli/TreeCountSegHeight. The relevant code can be found at https://github.com/sizhuoli/TreeCountSegHeight.

## References

[1] Lindner M , et al 2010. Climate change impacts, adaptive capacity, and vulnerability of European forest ecosystems. For Ecol Manage. 259:698–709.

[2] Brondizio ES , Settele J, Díaz S, Ngo H, Guèze M. 2019. *IPBES (2019): global assessment report on biodiversity and ecosystem services of the Intergovernmental Science-Policy Platform on Biodiversity and Ecosystem Services*.

[3] Canadell JG , Raupach MR. 2008. Managing forests for climate change mitigation. Science. 320:1456–1457. 18556550 10.1126/science.1155458

[4] Forster EJ , Healey JR, Dymond C, Styles D. 2021. Commercial afforestation can deliver effective climate change mitigation under multiple decarbonisation pathways. Nat Commun. 12:3831. 34158494 10.1038/s41467-021-24084-x PMC8219817

[5] *Forest Europe (2020)* . 2020. *State of Europe's forests*.

[6] Tomppo E , Gschwantner T, Lawrence M, McRoberts RE. 2010. National forest inventories. Dordrecht, Netherlands: Springer.

[7] Tomppo E , et al 2011. Designing and conducting a forest inventory—case: 9th national forest inventory of Finland. Dordrecht, Netherlands: Springer.

[8] Fischer C , Traub B. 2019. Swiss National Forest Inventory—methods and models of the fourth assessment. Cham, Switzerland: Springer.

[9] Schnell S , Kleinn C, Ståhl G. 2015. Monitoring trees outside forests: a review. Environ Monit Assess. 187:600. 26318320 10.1007/s10661-015-4817-7

[10] Fridman J , et al 2014. Adapting national forest inventories to changing requirements—the case of the Swedish National Forest Inventory at the turn of the 20th century. Silva Fennica. 48:1–29.

[11] Hansen MC , et al 2013. High-resolution global maps of 21st-century forest cover change. Science. 342:850–853. 24233722 10.1126/science.1244693

[12] Becker A , et al 2023. Country-wide retrieval of forest structure from optical and SAR satellite imagery with deep ensembles. ISPRS J Photogramm Remote Sens. 195:269–286.

[13] Astola H , Seitsonen L, Halme E, Molinier M, Lönnqvist A. 2021. Deep neural networks with transfer learning for forest variable estimation using sentinel-2 imagery in boreal forest. Remote Sens (Basel). 13:2392.

[14] Nord-Larsen T , Schumacher J. 2012. Estimation of forest resources from a country wide laser scanning survey and national forest inventory data. Remote Sens Environ. 119:148–157.

[15] Hufkens K , et al 2012. Linking near-surface and satellite remote sensing measurements of deciduous broadleaf forest phenology. Remote Sens Environ. 117:307–321.

[16] Berra EF , Gaulton R. 2021. Remote sensing of temperate and boreal forest phenology: a review of progress, challenges and opportunities in the intercomparison of in-situ and satellite phenological metrics. For Ecol Manage. 480:118663.

[17] Frolking S , et al 2009. Forest disturbance and recovery: a general review in the context of spaceborne remote sensing of impacts on aboveground biomass and canopy structure. J Geophys Res Biogeosci. 114: G00E02.

[18] Hermosilla T , Bastyr A, Coops NC, White JC, Wulder MA. 2022. Mapping the presence and distribution of tree species in Canada's forested ecosystems. Remote Sens Environ. 282:113276.

[19] Fassnacht FE , et al 2016. Review of studies on tree species classification from remotely sensed data. Remote Sens Environ. 186:64–87.

[20] Tomppo E , et al 2008. Combining national forest inventory field plots and remote sensing data for forest databases. Remote Sens Environ. 112:1982–1999.

[21] Turner W , et al 2015. Free and open-access satellite data are key to biodiversity conservation. Biol Conserv. 182:173–176.

[22] Ceccherini G , et al 2020. Abrupt increase in harvested forest area over Europe after 2015. Nature. 583:72–77. 32612223 10.1038/s41586-020-2438-y

[23] Palahí M , et al 2021. Concerns about reported harvests in European forests. Nature. 592:E15–E17. 33911265 10.1038/s41586-021-03292-x

[24] Fox JC , Ades PK, Bi H. 2001. Stochastic structure and individual-tree growth models. For Ecol Manage. 154:261–276.

[25] Biging GS , Dobbertin M. 1995. Evaluation of competition indices in individual tree growth models. Forest Science. 41:360–377.

[26] Rohner B , Waldner P, Lischke H, Ferretti M, Thürig E. 2018. Predicting individual-tree growth of central European tree species as a function of site, stand, management, nutrient, and climate effects. Eur J For Res. 137:29–44.

[27] Fritts H . 1976. Tree rings and climate. New York: Academic Press.

[28] Laubhann D , Sterba H, Reinds GJ, de Vries W. 2009. The impact of atmospheric deposition and climate on forest growth in European monitoring plots: an individual tree growth model. For Ecol Manage. 258:1751–1761.

[29] Baffetta F , Corona P, Fattorini L. 2011. Assessing the attributes of scattered trees outside the forest by a multi-phase sampling strategy. Forestry. 84:315–325.

[30] Schnell S , Altrell D, Ståhl G, Kleinn C. 2015. The contribution of trees outside forests to national tree biomass and carbon stocks—a comparative study across three continents. Environ Monit Assess. 187:4197. 25514855 10.1007/s10661-014-4197-4

[31] Krishnankutty CN , Thampi KB, Chundamannil M. 2008. Trees outside forests (TOF): a case study of the wood productionconsumption situation in Kerala. Int For Rev. 10:156–164.

[32] Smeets EMW , Faaij APC. 2007. Bioenergy potentials from forestry in 2050. Clim Change. 81:353–390.

[33] Skole DL , Mbow C, Mugabowindekwe M, Brandt MS, Samek JH. 2021. Trees outside of forests as natural climate solutions. Nat Clim Chang. 11:1013–1016.

[34] Drusch M , et al 2012. Sentinel-2: ESA's optical high-resolution mission for GMES operational services. Remote Sens Environ. 120:25–36.

[35] Wulder MA , et al 2019. Current status of Landsat program, science, and applications. Remote Sens Environ. 225:127–147.

[36] Zhang C , Zhou Y, Qiu F. 2015. Individual tree segmentation from LiDAR point clouds for urban forest inventory. Remote Sens (Basel). 7:7892–7913.

[37] Hyyppä J , et al 2008. Review of methods of small-footprint airborne laser scanning for extracting forest inventory data in boreal forests. Int J Remote Sens. 29:1339–1366.

[38] Budei BC , St-Onge B, Hopkinson C, Audet F-A. 2018. Identifying the genus or species of individual trees using a three-wavelength airborne LiDAR system. Remote Sens Environ. 204:632–647.

[39] Dalagnol R , et al 2022. Canopy palm cover across the Brazilian Amazon forests mapped with airborne LiDAR data and deep learning. Remote Sens Ecol Conserv. 8:601–614.

[40] Hyyppa J , Kelle O, Lehikoinen M, Inkinen M. 2001. A segmentation-based method to retrieve stem volume estimates from 3-D tree height models produced by laser scanners. IEEE Trans Geosci Remote Sens. 39:969–975.

[41] Kauranne T , et al 2017. Airborne laser scanning based forest inventory: comparison of experimental results for the Perm Region, Russia and prior results from Finland. Forests. 8:72.

[42] Oehmcke, S. et al 2022. Deep learning based 3D point cloud regression for estimating forest biomass. Proceedings of the 30th International Conference on Advances in Geographic Information Systems, 1–4. ACM.

[43] Freudenberg M , Magdon P, Nölke N. 2022. Individual tree crown delineation in high-resolution remote sensing images based on U-Net. Neural Comput Appl. 34:22197–22207.

[44] Braga JRG , et al 2020. Tree crown delineation algorithm based on a convolutional neural network. Remote Sens (Basel). 12:1288.

[45] Wagner FH , et al 2018. Individual tree crown delineation in a highly diverse tropical forest using very high resolution satellite images. ISPRS J Photogramm Remote Sens. 145:362–377.

[46] Lassalle G , Ferreira MP, la Rosa LEC, de Souza Filho CR. 2022. Deep learning-based individual tree crown delineation in mangrove forests using very-high-resolution satellite imagery. ISPRS J Photogramm Remote Sens. 189:220–235.

[47] Osco LP , et al 2020. A convolutional neural network approach for counting and geolocating citrus-trees in UAV multispectral imagery. ISPRS J Photogramm Remote Sens. 160:97–106.

[48] Mubin NA , Nadarajoo E, Shafri HZM, Hamedianfar A. 2019. Young and mature oil palm tree detection and counting using convolutional neural network deep learning method. Int J Remote Sens. 40:7500–7515.

[49] Wang Y , Zhu X, Wu B. 2019. Automatic detection of individual oil palm trees from UAV images using HOG features and an SVM classifier. Int J Remote Sens. 40:7356–7370.

[50] Brandt M , et al 2020. An unexpectedly large count of trees in the West African Sahara and Sahel. Nature. 587:78–82. 33057199 10.1038/s41586-020-2824-5

[51] Wang Y , et al 2019. In situ biomass estimation at tree and plot levels: what did data record and what did algorithms derive from terrestrial and aerial point clouds in boreal forest. Remote Sens Environ. 232:111309.

[52] Salum RB , et al 2020. Improving mangrove above-ground biomass estimates using LiDAR. Estuar Coast Shelf Sci. 236:106585.

[53] Jucker T , et al 2017. Allometric equations for integrating remote sensing imagery into forest monitoring programmes. Glob Chang Biol. 23:177–190. 27381364 10.1111/gcb.13388 PMC6849852

[54] Falk T , et al 2019. U-Net: deep learning for cell counting, detection, and morphometry. Nat Methods. 16:67–70. 30559429 10.1038/s41592-018-0261-2

[55] Lugagne J-B , Lin H, Dunlop MJ. 2020. DeLTA: automated cell segmentation, tracking, and lineage reconstruction using deep learning. PLoS Comput Biol. 16:e1007673. 10.1371/journal.pcbi.1007673 PMC7153852 32282792

[56] Long J , Shelhamer E, Darrell T. 2015. Fully convolutional networks for semantic segmentation. 2015 IEEE Conference on Computer Vision and Pattern Recognition (CVPR), 3431–3440; IEEE. 10.1109/TPAMI.2016.2572683 27244717

[57] Farabet C , Couprie C, Najman L, LeCun Y. 2013. Learning hierarchical features for scene labeling. IEEE Trans Pattern Anal Mach Intell. 35:1915–1929. 23787344 10.1109/TPAMI.2012.231

[58] Pinheiro P , Collobert R. 2014. Recurrent convolutional neural networks for scene labeling. Proceedings of the 31st International Conference on Machine Learning, 82–90, PMLR.

[59] Boominathan L , Kruthiventi SSS, Babu RV. 2016. Crowdnet: a deep convolutional network for dense crowd counting. Proceedings of the 24th ACM International Conference on Multimedia, ACM.

[60] Zhang C , Li H, Wang X, Yang X. 2015. Cross-scene crowd counting via deep convolutional neural networks. Proceedings of the IEEE Conference on Computer Vision and Pattern Recognition, CVPR.

[61] Amirkolaee HA , Arefi H. 2019. Height estimation from single aerial images using a deep convolutional encoder-decoder network. ISPRS J Photogramm Remote Sens. 149:50–66.

[62] Potapov P , et al 2021. Mapping global forest canopy height through integration of GEDI and Landsat data. Remote Sens Environ. 253:112165.

[63] Lang N , Schindler K, Wegner JD. 2019. Country-wide high-resolution vegetation height mapping with Sentinel-2. Remote Sens Environ. 233:111347.

[64] Ronneberger O , Fischer P, Brox T. 2015. U-Net: convolutional networks for biomedical image segmentation. International Conference on Medical Image Computing and Computer-Assisted Intervention, 234–241.

[65] Oktay O . et al 2018. Attention U-Net: learning where to look for the pancreas.

[66] European Environment Agency (EEA) . 2018. Copernicus land monitoring service. 10.1007/BF02986943 24234300

[67] Kobayashi K , Salam MU. 2000. Comparing simulated and measured values using mean squared deviation and its components. Agron J. 92:345–352.

[68] Nord-Larsen T , Johannsen VK. 2016. *Danish National Forest Inventory. Design and calculations*.

[69] Nord-Larsen T , Johannsen VK, Riis-Nielsen T, Thomsen, IM, Jørgensen BB. 2020. *Skovstatistik 2018: forest statistics 2018. (2 udg.)*.

[70] Metsäkeskus S . 2018. *Puustotulkintakoealojen Maastotyöohje*.

[71] Li W , et al 2020. High-resolution mapping of forest canopy height using machine learning by coupling ICESat-2 LiDAR with Sentinel-1, Sentinel-2 and Landsat-8 data. Int J Appl Earth Obs Geoinf. 92:102163.

[72] Murthy K . et al 2014. SkySat-1: very high-resolution imagery from a small satellite. Proceeding SPIE 9241, Sensors, Systems, and Next-Generation Satellites XVIII, 92411E.

[73] Saunier S , et al 2022. SkySat data quality assessment within the EDAP framework. Remote Sens (Basel). 14:1646.

[74] Bhushan S , Shean D, Alexandrov O, Henderson S. 2021. Automated digital elevation model (DEM) generation from very-high-resolution Planet SkySat triplet stereo and video imagery. ISPRS J Photogramm Remote Sens. 173:151–165.

[75] Mugabowindekwe M , et al 2022. Nation-wide mapping of tree-level aboveground carbon stocks in Rwanda. Nat Clim Chang. 13:91–97. 36684409 10.1038/s41558-022-01544-w PMC9845119

[76] Campbell MJ , et al 2020. A multi-sensor, multi-scale approach to mapping tree mortality in woodland ecosystems. Remote Sens Environ. 245:111853.

[77] McMahon SM , Arellano G, Davies SJ. 2019. The importance and challenges of detecting changes in forest mortality rates. Ecosphere. 10:e02615.

[78] Song H , Kim M, Park D, Shin Y, Lee J-G. 2020. Learning from noisy labels with deep neural networks: a survey.

[79] Hafiz AM , Bhat GM. 2020. A survey on instance segmentation: state of the art. Int J Multimed Inf Retr. 9:171–189.

[80] He K , Gkioxari G, Dollar P, Girshick R. 2020. Mask R-CNN. IEEE Trans Pattern Anal Mach Intell. 42:386–397. 29994331 10.1109/TPAMI.2018.2844175

[81] Li Y , Qi H, Dai J, Ji X, Wei Y. 2017. Fully convolutional instance-aware semantic segmentation. 2017 IEEE Conference on Computer Vision and Pattern Recognition (CVPR), 4438–4446, IEEE.

[82] Bai M , Urtasun R. 2016. Deep watershed transform for instance segmentation.

[83] Ke L , Tai Y-W, Tang C-K. 2021. Deep occlusion-aware instance segmentation with overlapping bilayers.

[84] Habib AF , Kim E-M, Kim C-J. 2007. New methodologies for true orthophoto generation. Photogramm Eng Remote Sensing. 73:25–36.

[85] Wang P . et al 2018. Understanding convolution for semantic segmentation. 2018 IEEE Winter Conference on Applications of Computer Vision (WACV), 1451–1460, IEEE.

[86] Zhang Y , Zhou D, Chen S, Gao S, Ma Y. 2016. Single-image crowd counting via multi-column convolutional neural network. 2016 IEEE Conference on Computer Vision and Pattern Recognition (CVPR), 589–597, IEEE.

[87] Ioffe S , Szegedy C. 2015. Batch normalization: accelerating deep network training by reducing internal covariate shift.

[88] Kingma DP , Ba, J. 2014. Adam: a method for stochastic optimization.

[89] Tversky A . 1977. Features of similarity. Psychol Rev. 84:327–352.

[90] Milletari F , Navab N, Ahmadi S.-A. 2016. V-Net: fully convolutional neural networks for volumetric medical image segmentation. 2016 Fourth International Conference on 3D Vision (3DV), 565–571, IEEE.

[91] Salehi SSM , Erdogmus D, Gholipour A. 2017. Tversky loss function for image segmentation using 3D fully convolutional deep networks. International Workshop on Machine Learning in Medical Imaging; 379–387.

[92] Shorten C , Khoshgoftaar TM. 2019. A survey on image data augmentation for deep learning. J Big Data. 6:60. 10.1186/s40537-021-00492-0 PMC8287113 34306963

[93] Igel C , Oehmcke S. 2022. Remember to correct the bias when using deep learning for regression!

[94] Chalmers RP , Adkins MC. 2020. Writing effective and reliable Monte Carlo simulations with the SimDesign package. Quant Method Psychol. 16:248–280.

[95] Sigal MJ , Chalmers RP. 2016. Play it again: teaching statistics with Monte Carlo simulation. J Stat Educ. 24:136–156.

