# Supplementary material for: Deep learning enables image-based tree counting, crown segmentation, and height prediction at national scale
Source: PNAS Nexus. 2023 Mar 9;2(4):pgad076. doi: 10.1093/pnasnexus/pgad076 (PMC10096914; doi:10.1093/pnasnexus/pgad076)
Supplement: pgad076_Supplementary_Data [file pgad076_supplementary_data.docx]

**Supplementary Materials**

Table S1. **Performance comparison between separate models trained using different spectral band compositions.** Model M2 was trained using canopy height maps predicted from aerial images. See visual examples in Figure S1.

| Model | Band composition | F1-score | Precision | Recall | Mean IoU | Relative bias for counts (%) |
| --- | --- | --- | --- | --- | --- | --- |
| M1 | RGB + NIR + NDVI + CHM | 0.77 | 0.96 | 0.69 | 0.72 | 10.3 |
| M2 | RGB + NIR + NDVI + CHM (prediction) | 0.73 | 0.95 | 0.64 | 0.69 | 21.8 |
| M3 | RGB + NIR + NDVI | 0.77 | 0.96 | 0.69 | 0.72 | -11.5 |
| M4 | RGB + NIR | 0.76 | 0.96 | 0.67 | 0.71 | -12.2 |
| M5 | RGB | 0.73 | 0.96 | 0.64 | 0.69 | 17.1 |
| M6 | GB + NIR | 0.73 | 0.96 | 0.64 | 0.69 | -14.4 |
| M7 | NIR | 0.71 | 0.95 | 0.62 | 0.67 | -3.4 |


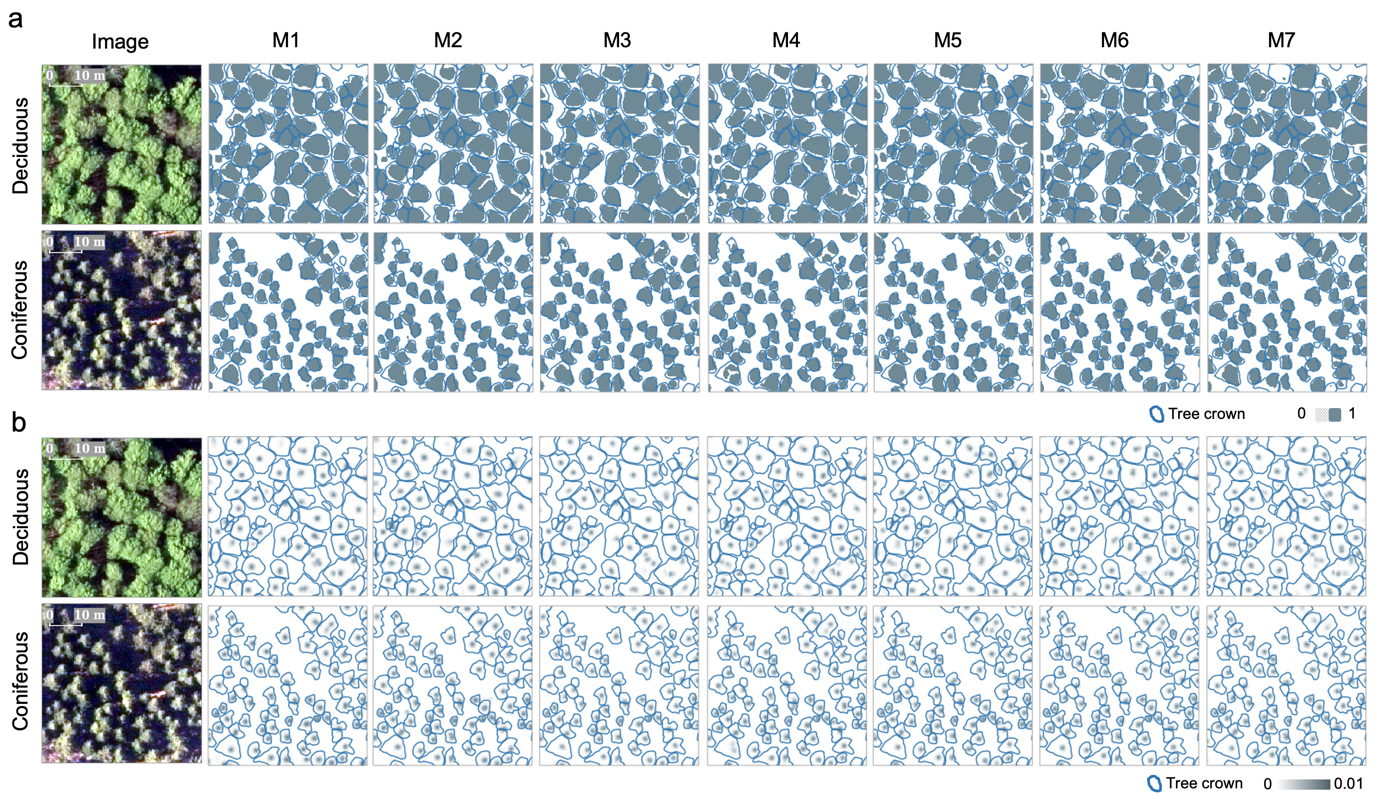


Figure S1. **Performance comparison between separate models trained using different spectral band compositions.** Evaluation on the whole testing data using the models described in Table S1. **a,** Individual tree crown segmentation examples from deciduous forest and coniferous forest, overlaid with the manual delineations (thin blue lines). **b,** Tree counting examples from deciduous forest and coniferous forest, overlaid with the manual delineations (thin blue lines).


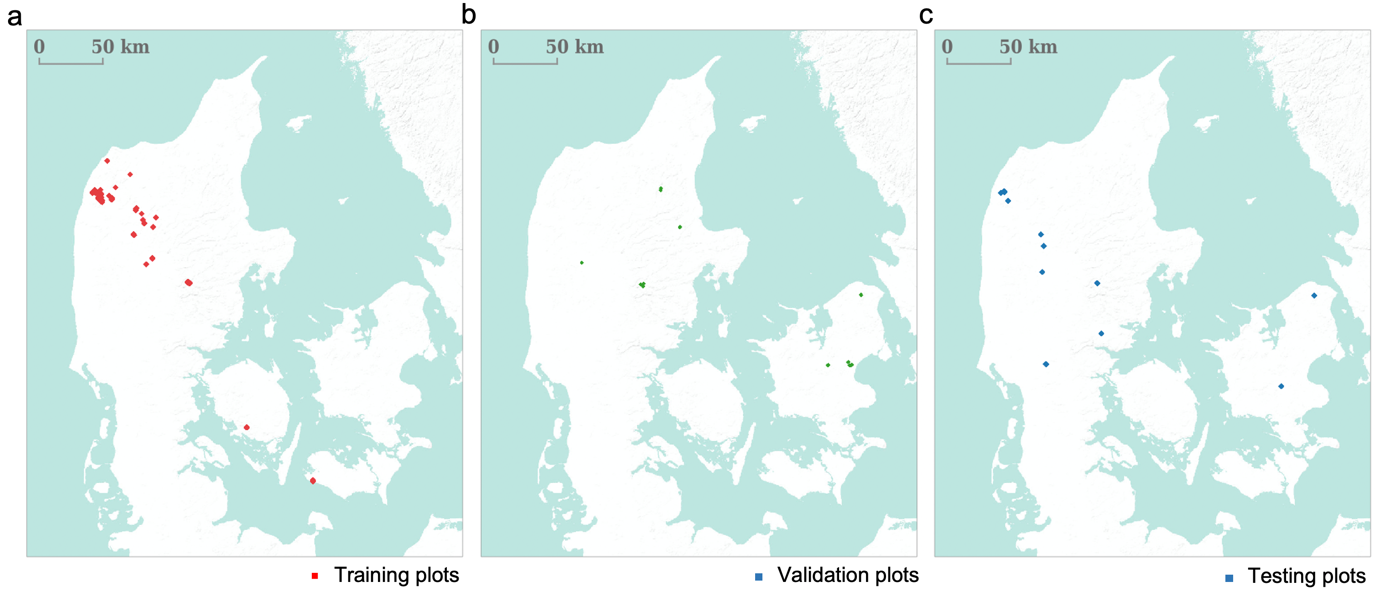


Figure S2. **Location of the training, validation, and testing data for the individual tree counting and crown segmentation model. a,** Individual tree crown delineations used for training the neural network, containing 19,771 individual trees in 75 sample plots of various sizes. **b,** Individual tree crown delineations used for model selection and hyper-parameter tuning, containing 2,016 individual trees in 25 sample plots of various sizes. **c,** Individual tree crown delineations used for evaluating the neural network, containing 2,679 individual trees in 25 sample plots of varying sizes.


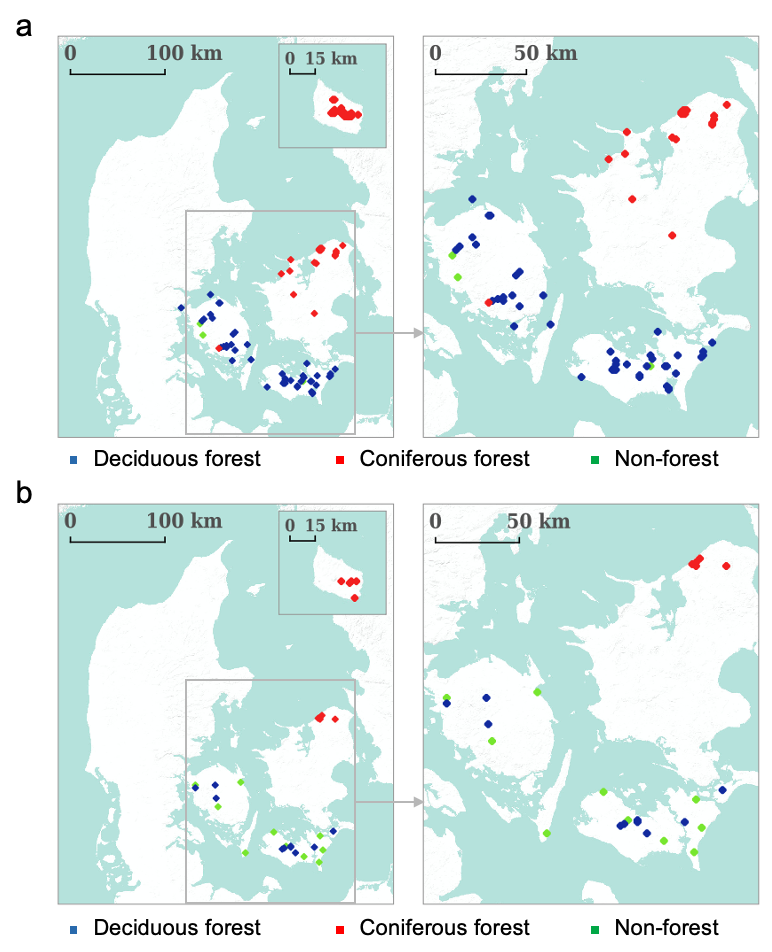


Figure S3. **Location of the training and testing data for the canopy / tree height prediction model. a,** Data used for training the neural network, containing 4.5 k ha of aerial images and canopy height maps from deciduous forest, 4.5 k ha from coniferous forest, and 0.3 k ha from non-forest areas. **b,** The standalone testing data: randomly sampled data used for evaluating the neural network, containing 1 k ha of aerial images and canopy height maps from deciduous forest, coniferous forest, non-forest areas, respectively.


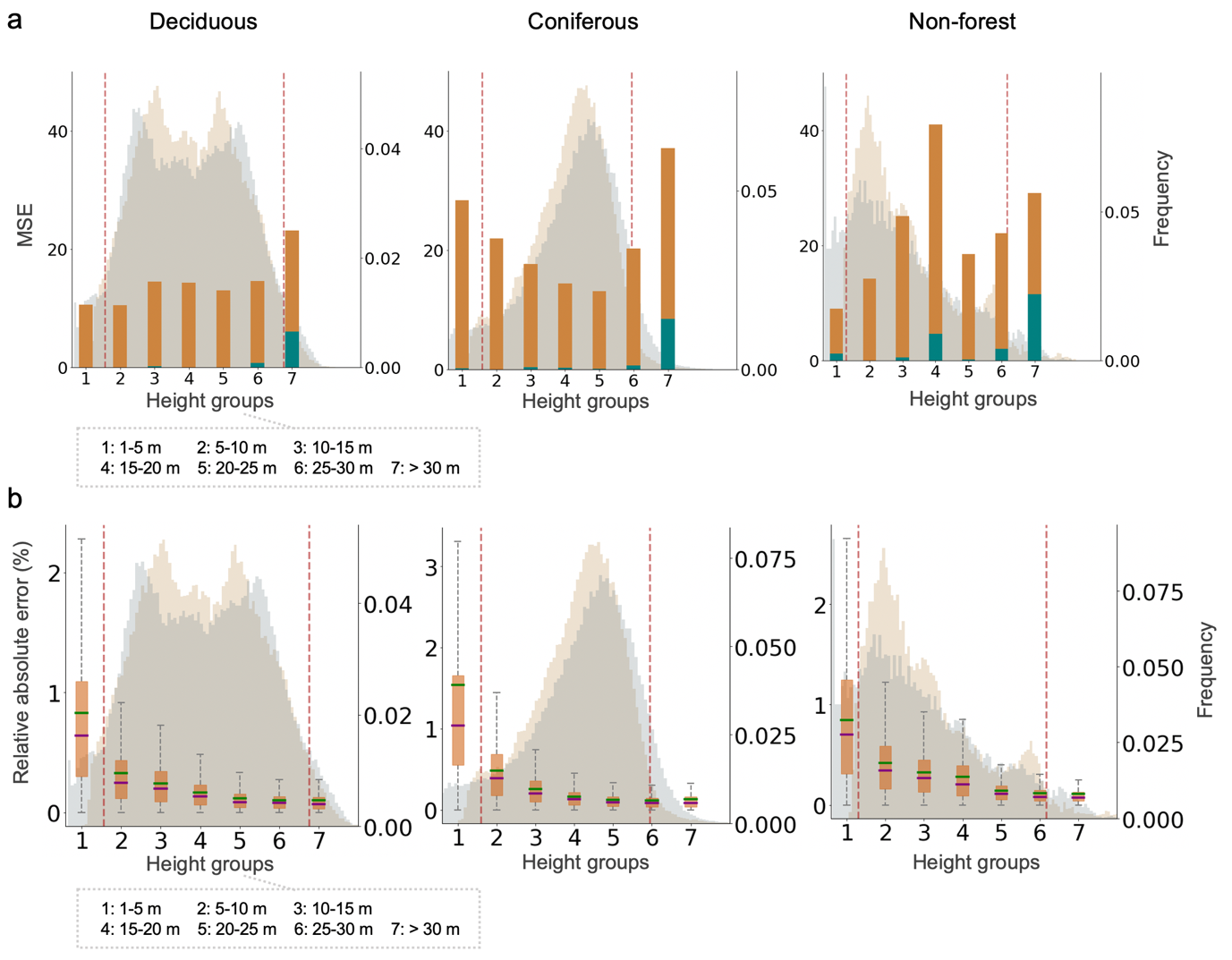


Figure S4. **Evaluation of individual tree height prediction. a,** Mean squared errors of the individual tree height prediction, decomposed into squared bias and mean squared variation. **b,** Relative absolute errors (similar as Figure 4b but using relative MAE instead of MAE) of the individual tree height prediction, grouped in 5 m height intervals, with the predicted and reference height distributions in the background.


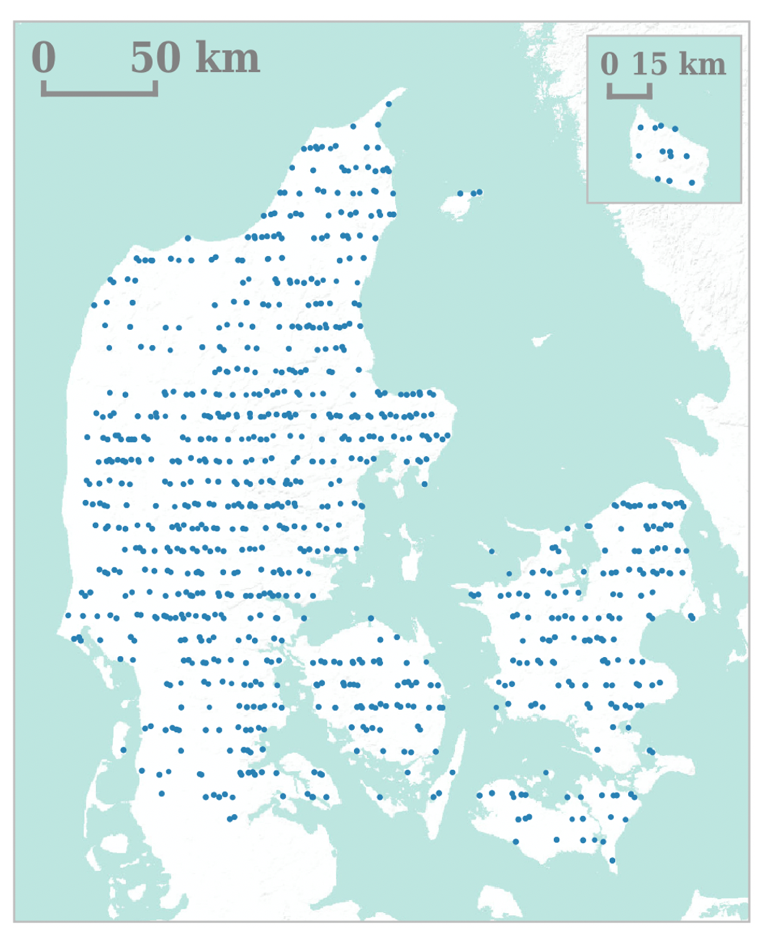


Figure S5. **The tree count evaluation against NFI data.** Locations of the 2563 NFI field plots used for the evaluation.


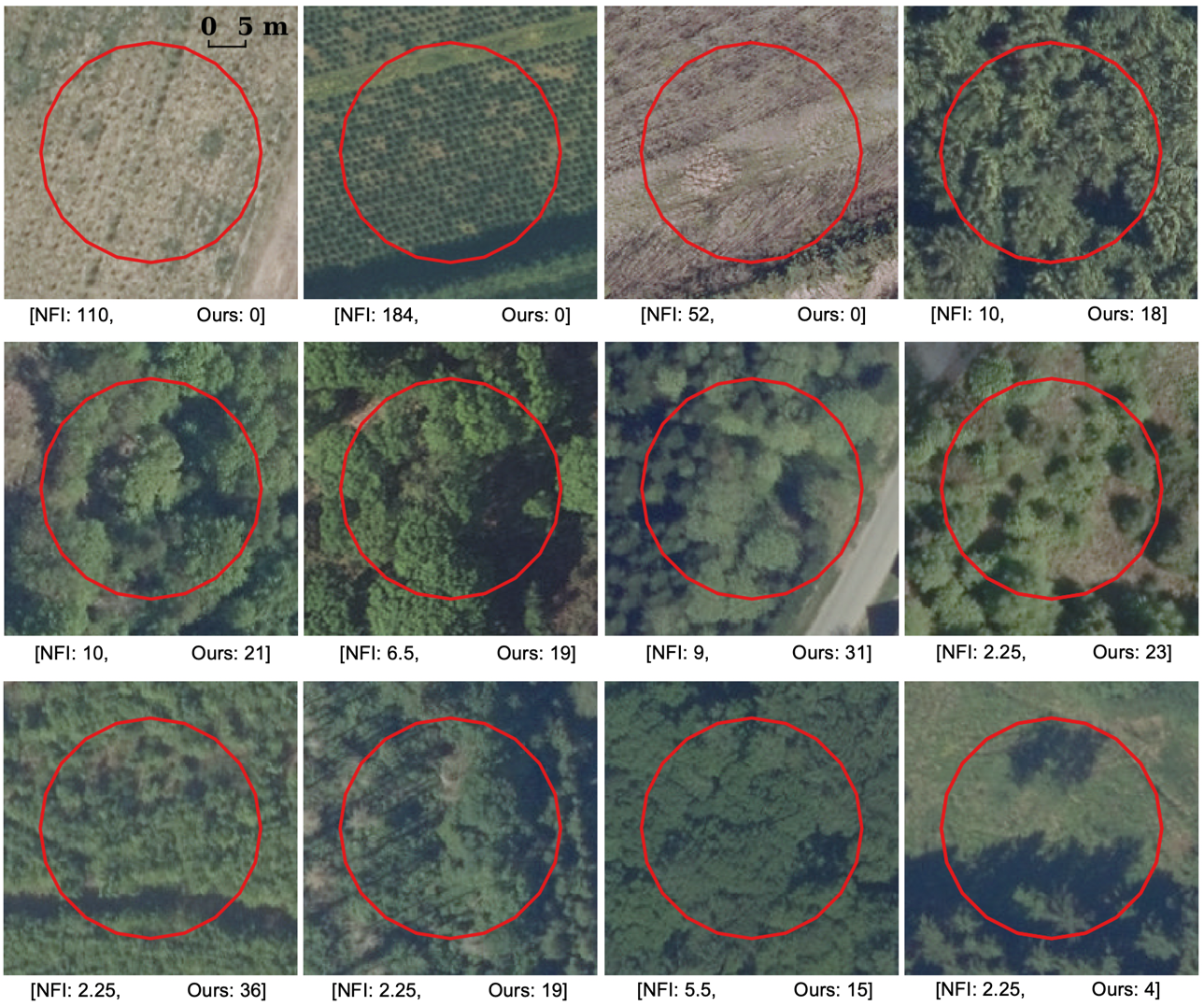


Figure S6. **Predicted tree counts compared against the NFI tree counts for all trees taller than 1.3 m.** Smaller trees (dbh < 40 m) not exhaustively measured on the field were extrapolated by assuming an even distribution of stems.


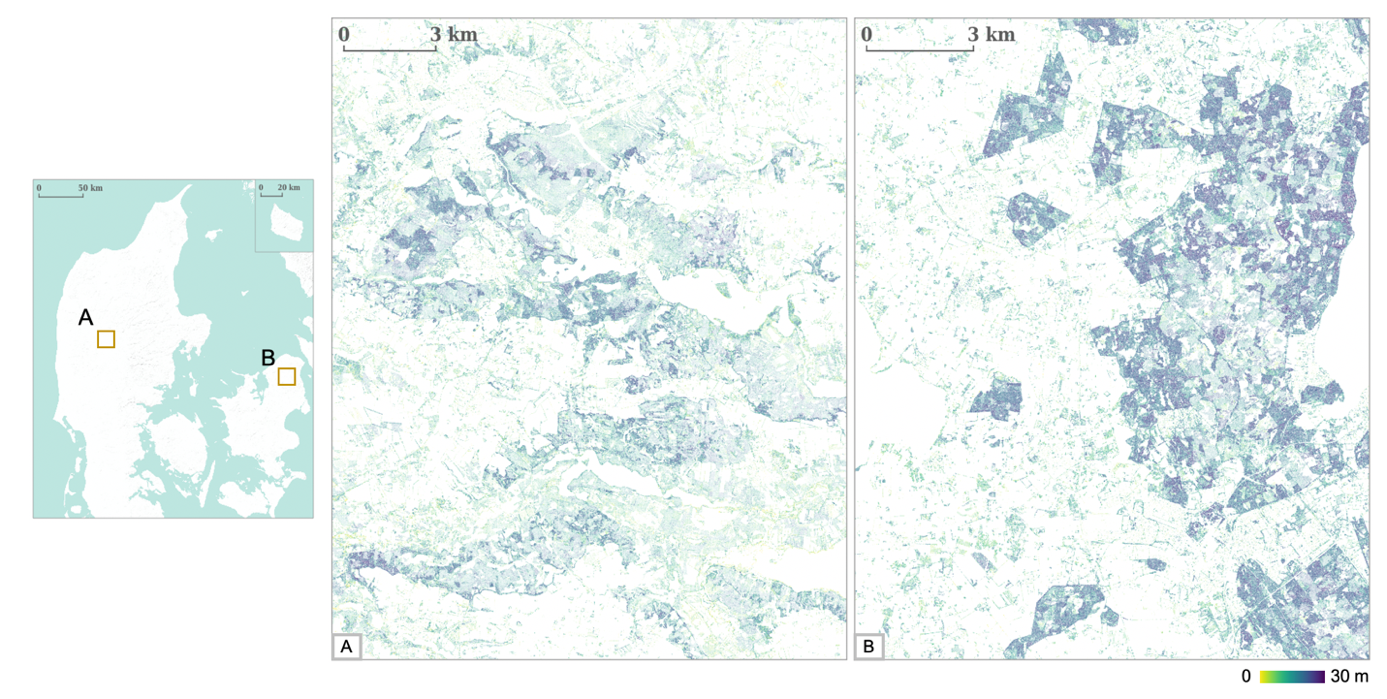


Figure S7. **Large-scale examples showing the individual tree crown segmentation results colored by height predictions.**


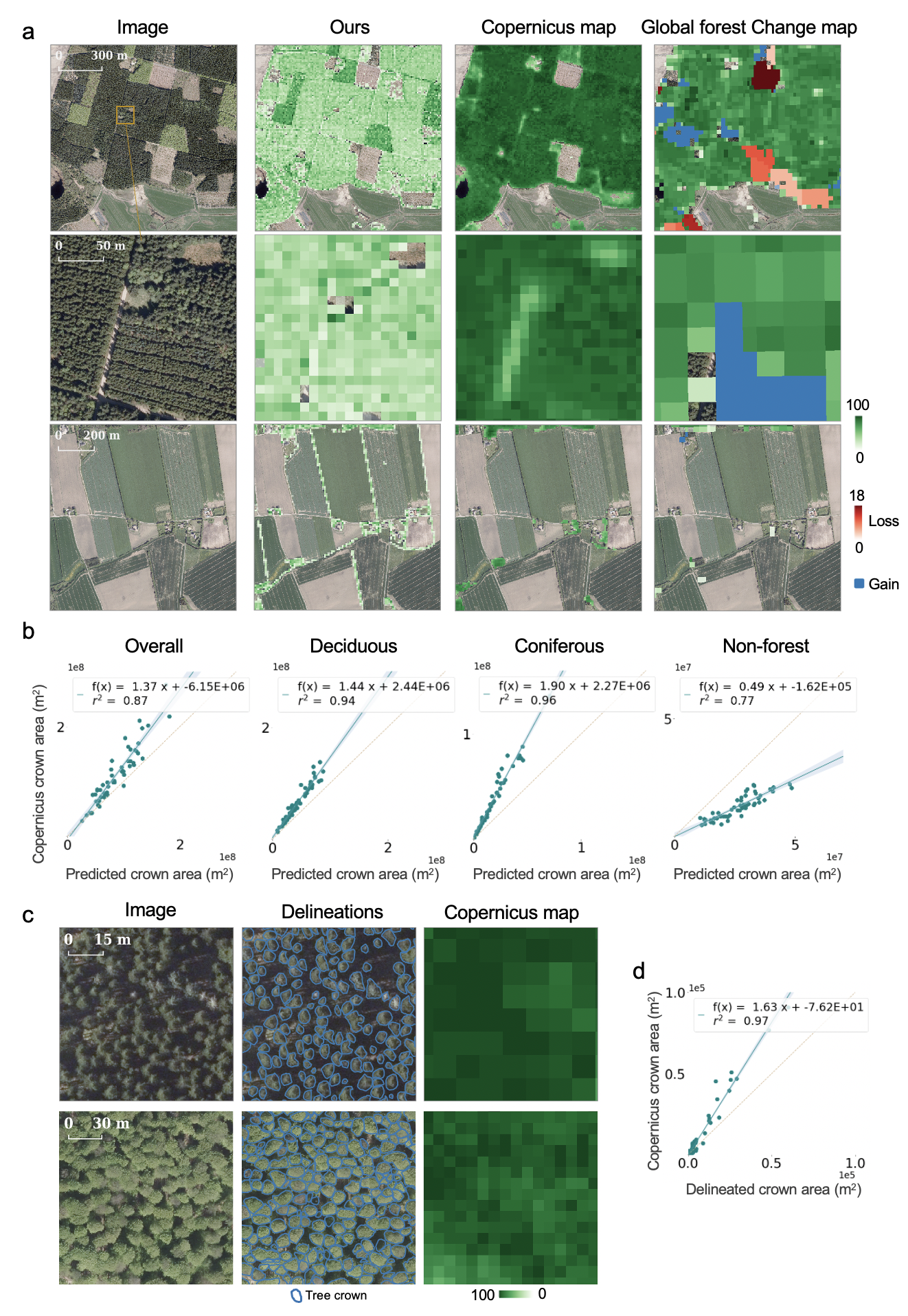


Figure S8. **Comparison of the tree crown cover with the Copernicus tree cover map** ^1^ **and the global forest change map** ^2^**. a,** Examples from forest and non-forest areas. Here, the percentage tree cover is shown in green colors. The losses are shown in red colors with higher values denoting losses in more recent years (starting from 2000) and the gains are shown in blue. **b,** Country-wide comparison of the predicted crown area with the Copernicus tree / crown cover for deciduous, coniferous, non-forest, and overall. Here, each point in the scatter plot represents a zone in Denmark. **c,** Examples comparing the manually delineated individual tree crowns (thin blue lines) and the Copernicus tree cover map for deciduous and coniferous forest. **d,** Comparison between the delineated crown area and the Copernicus crown area using the whole training dataset.

Table S2. **Comparison between total tree crown area in Denmark obtained from our results and the Copernicus tree cover map (2018)** ^1^**.**

| Forest / Landscape type | Total tree crown area (ha) | |
| --- | --- | --- |
|  | Ours | Copernicus map |
| Deciduous forest | 233,720 | 348,529 |
| Coniferous forest | 92,352 | 187,388 |
| Non-forest | 141,186 | 70,118 |
| Total | 467,257 | 606,035 |


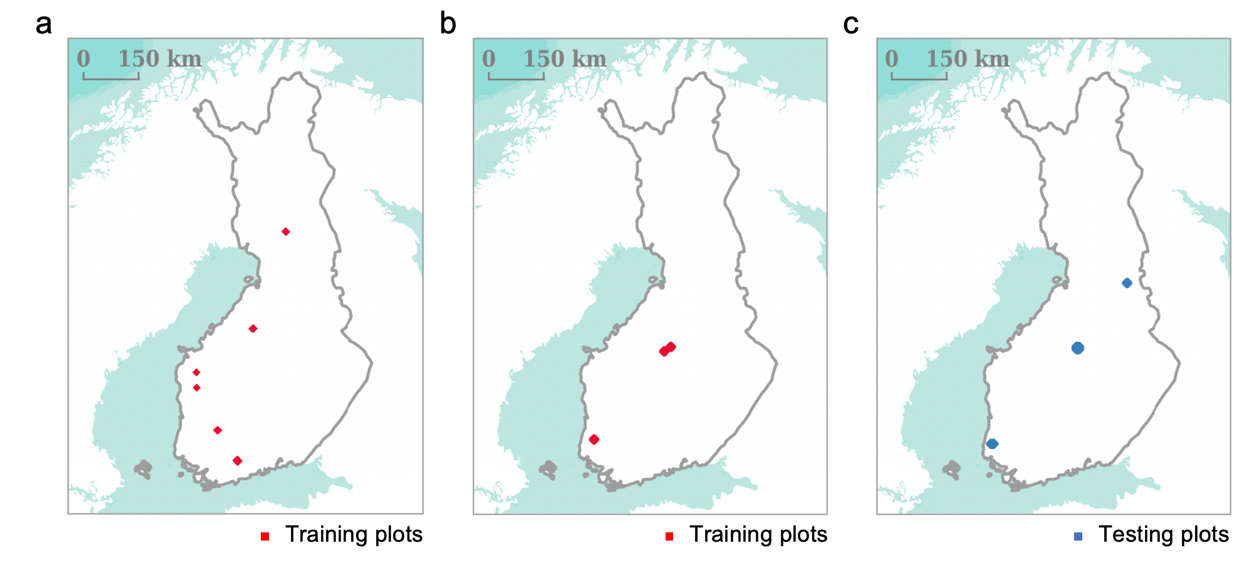


Figure S9. **Location of the training and testing data for transfer learning in Finland. a,** Individual tree crown delineations used for fine-tuning the pre-trained counting and crown segmentation model, containing 4,773 individual trees in 19 sample plots of varying sizes. **b,** Aerial images and the corresponding canopy height maps (10.7 k ha) used for fine-tuning the pre-trained canopy height prediction model. **c,** Aerial images and the corresponding canopy height maps (21.7 k ha) used for evaluating the individual tree height predictions.


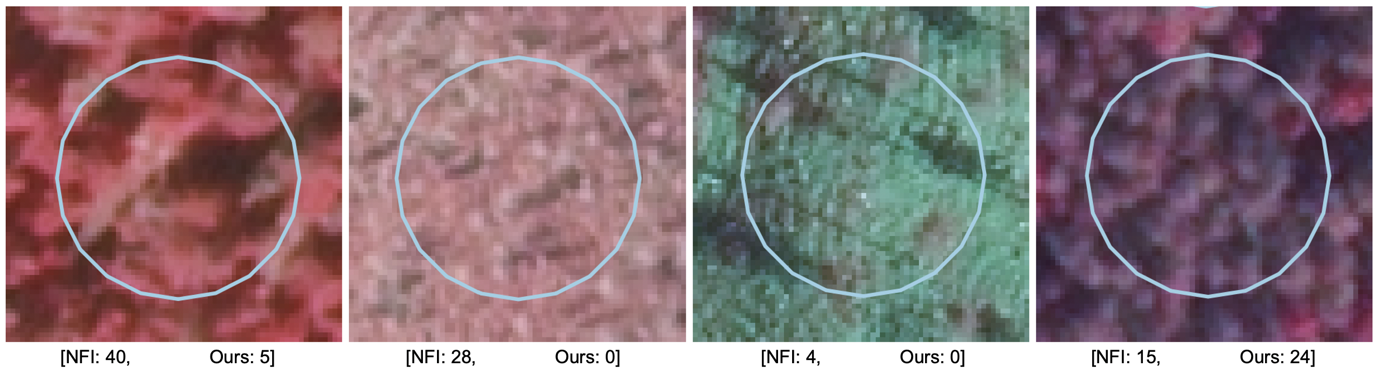


Figure S10. **Examples of tree count predictions evaluated against the NFI plot data (12.62 m radius) with high bias.** The NFI tree counts represent the number of trees taller than 1.3 m measured in each plot on the field.


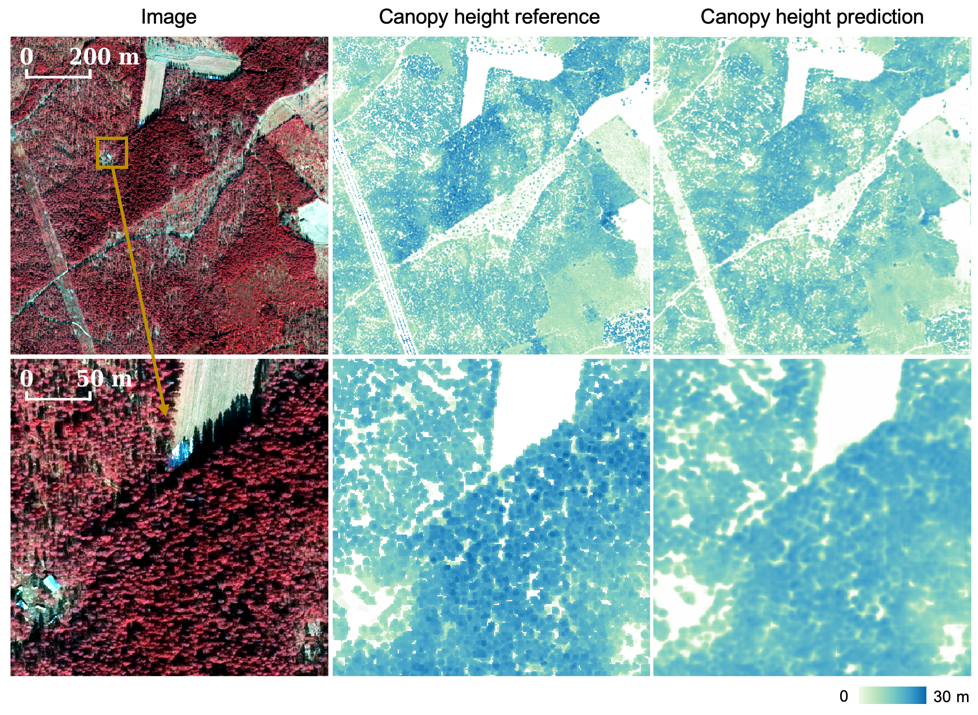


Figure S11. **Predicted canopy heights compared against reference canopy heights.** The second row displays the zoomed-in areas from the first row.


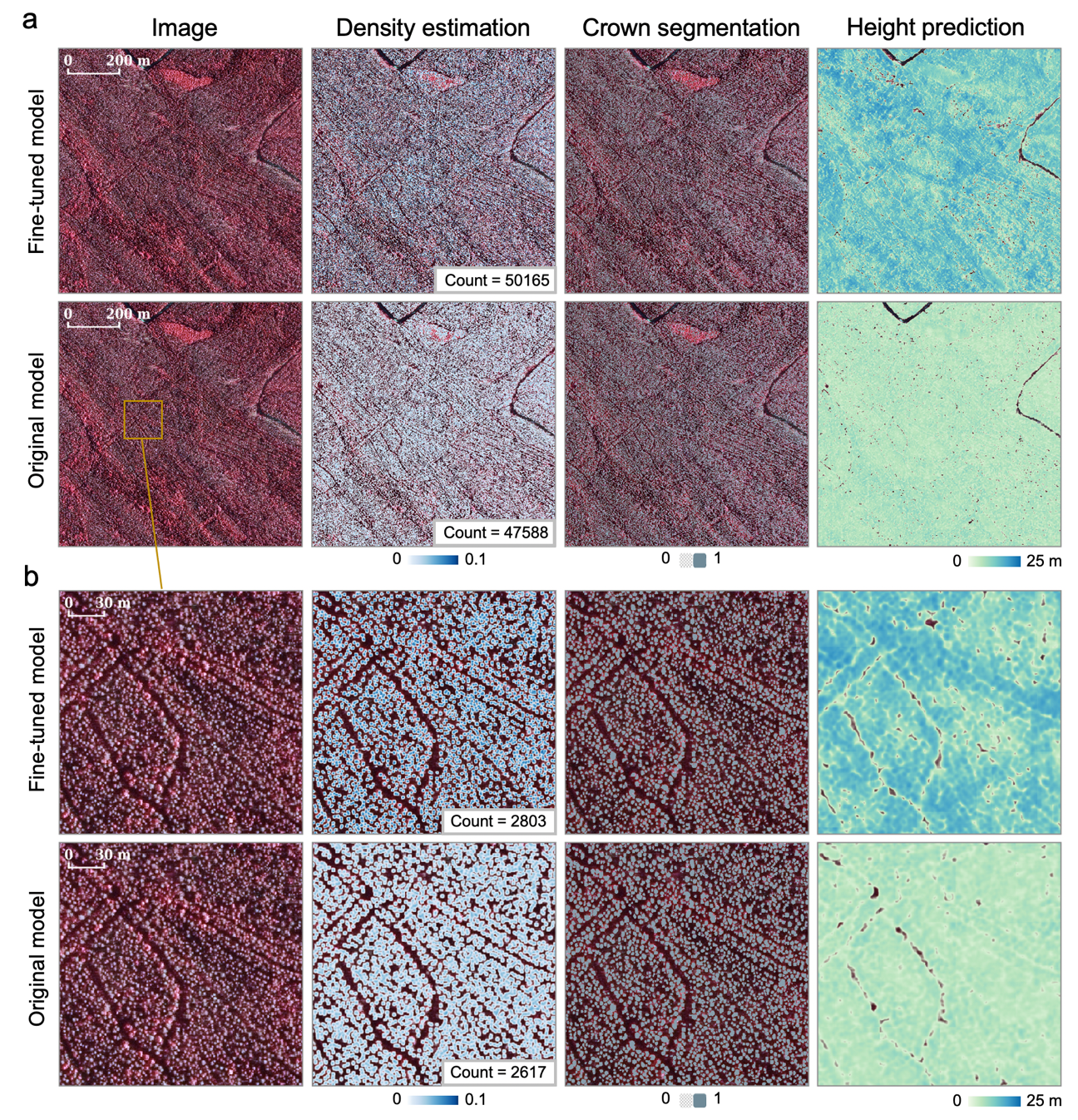


Figure S12. **Performance enhancement in Finland via transfer learning.** Comparison between results obtained using the original model trained with data from Denmark and the fine-tuned model trained with extra data from Finland. **a,** Large-scale examples showing the counting, crown segmentation, and height prediction results from the original model and the fine-tuned model. **b,** Detailed examples showing the results from the original model and the fine-tuned model.


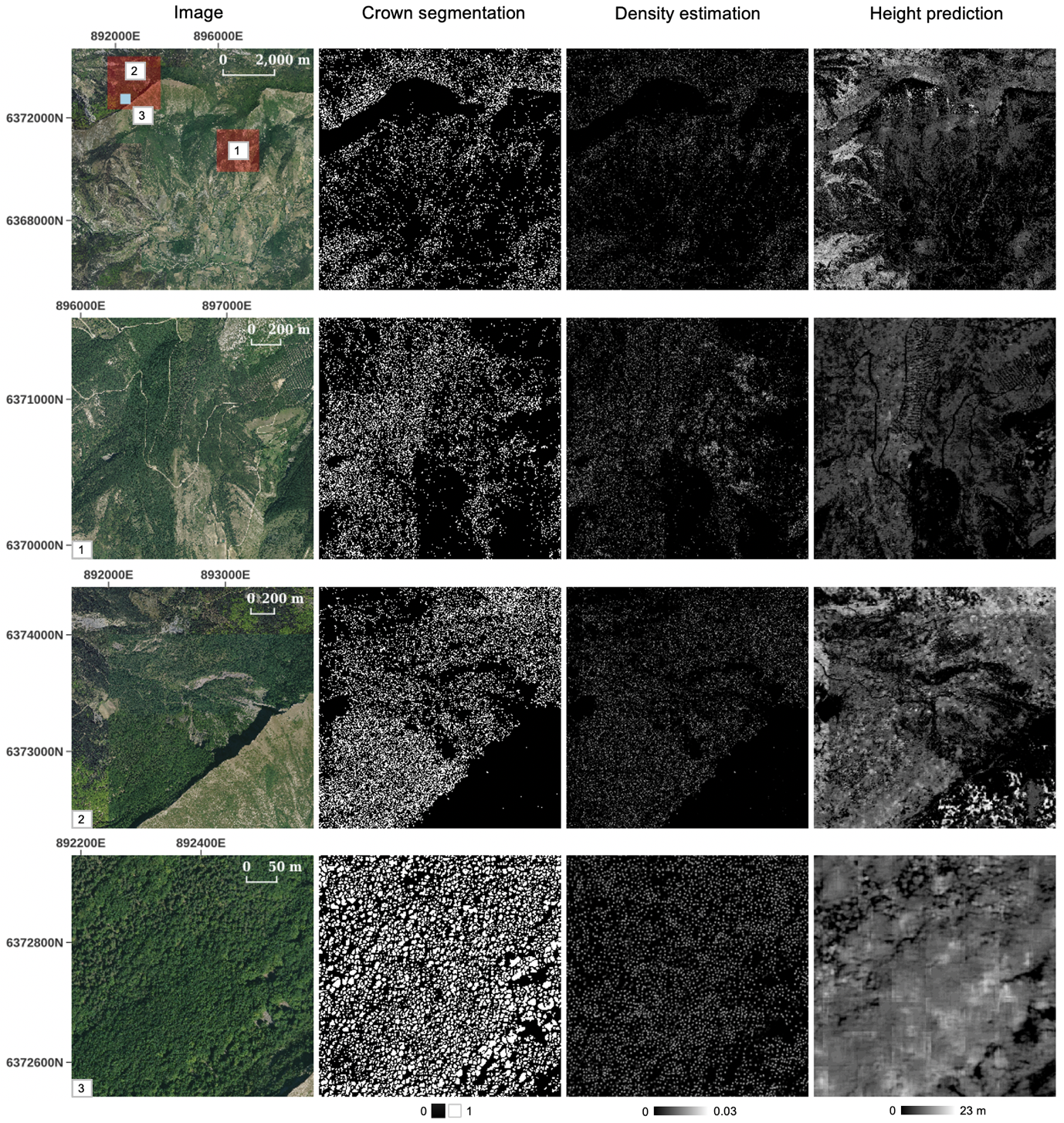


Figure S13. **Application of the models trained using aerial images from Denmark to aerial images from France (Baronnies Provençales Regional Nature Park).** The second, third and fourth row display the zoomed-in areas from the first row. The projection is EPSG 2154 – RGF93/Lambert-93, and the spatial resolution is 20 cm.


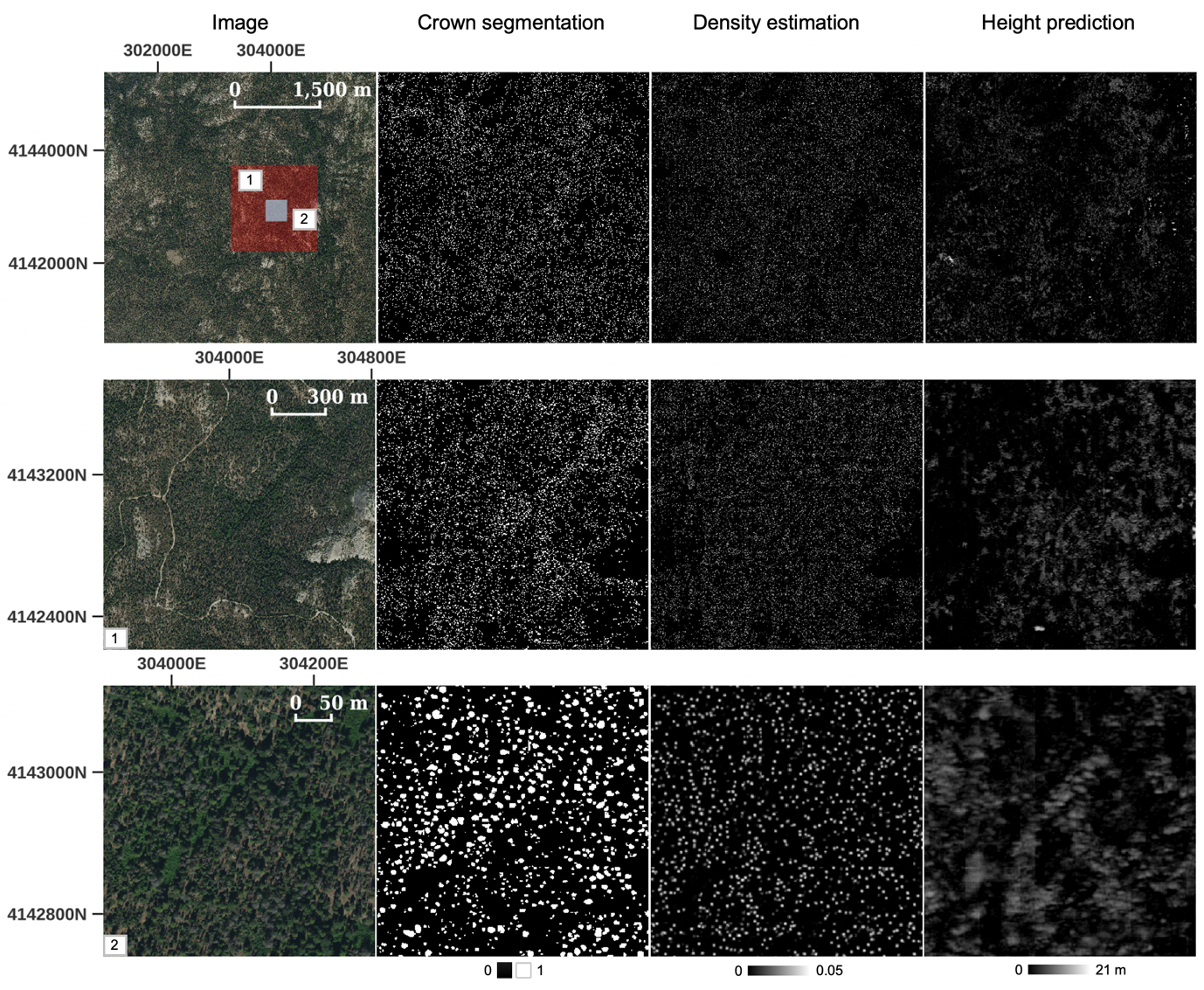


Figure S14. **Application of the models trained using aerial images from Denmark to aerial images from USA (Sierra National Forest).** The second and third row display the zoomed-in areas from the first row. The projection is EPSG 26911 – NAD83/UTM zone 11N, and the spatial resolution is 60 cm.


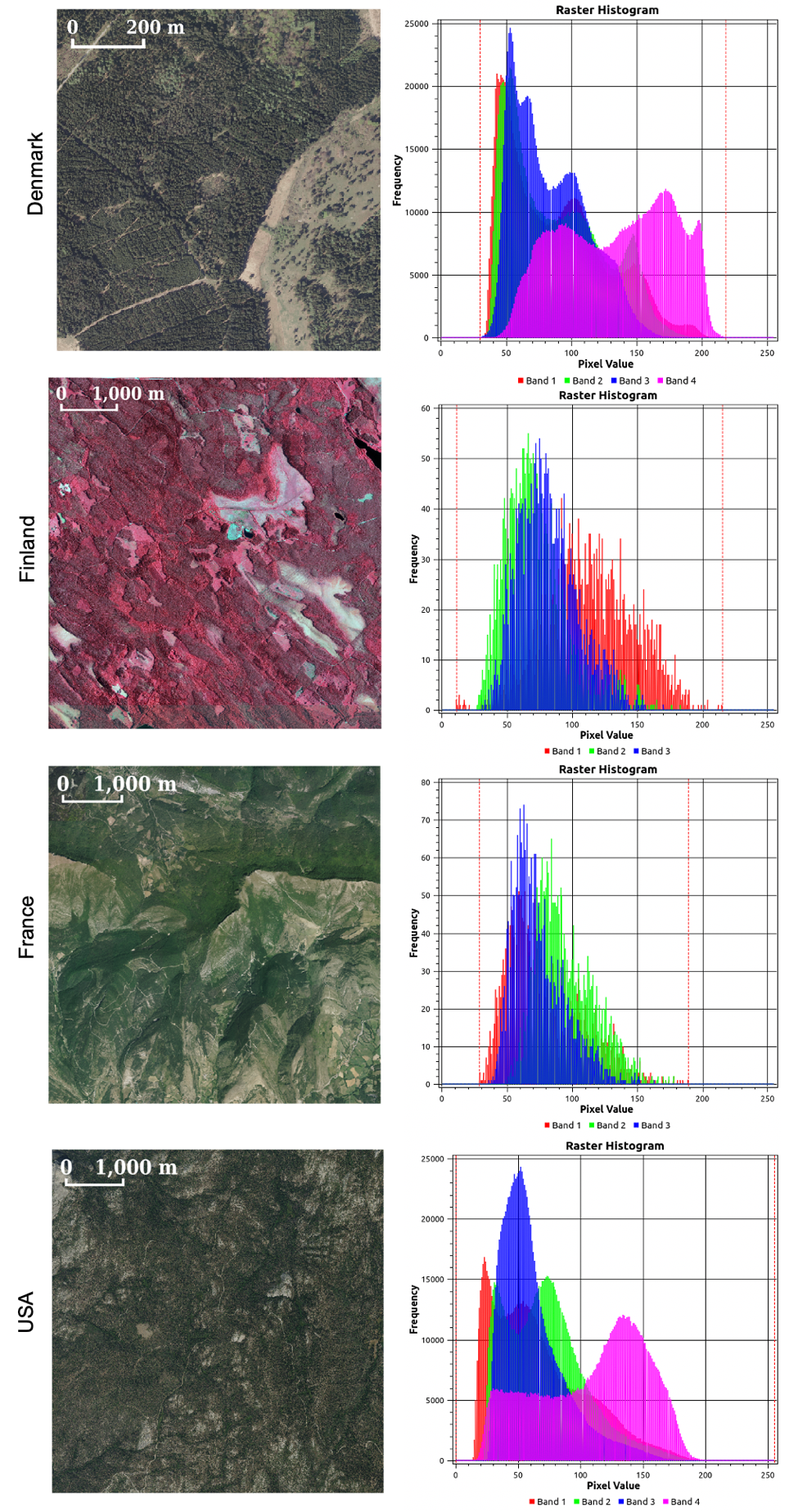


Figure S15. **Comparisons between distributions of input image pixel value intensities from different datasets.** Aerial images and the corresponding pixel value intensity distributions from the four datasets involved in this study, including Denmark (RGB+NIR), Finland (GB+NIR), France (RGB), and USA (RGB+NIR), shown column-wise. All samples were taken from forest areas.


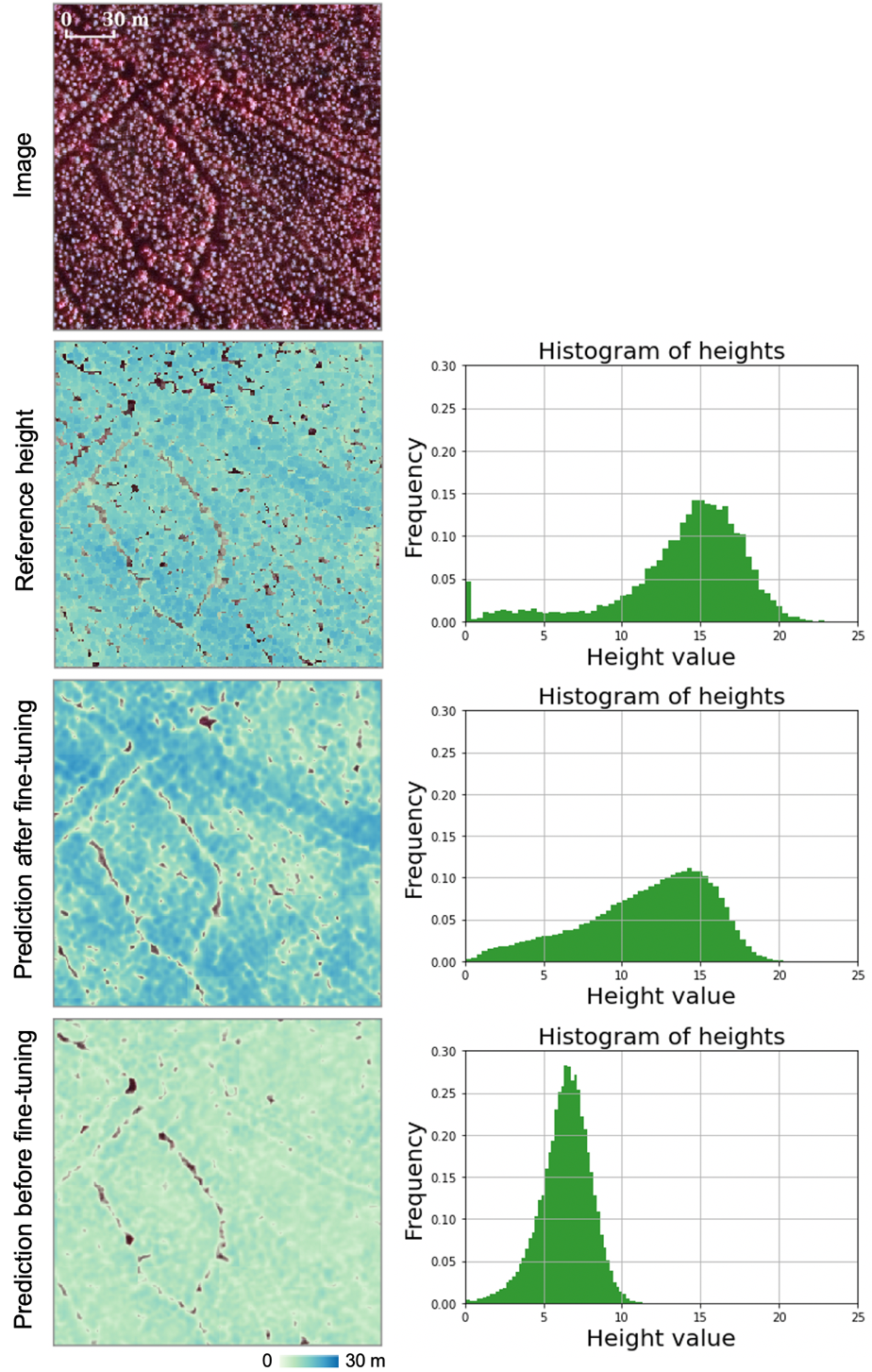


Figure S16. **Histograms of reference heights (second row), predicted heights from the fine-tuned model (third row), and predicted heights from the untuned model (forth row).** The Finish data is used here for illustration, See also Figure S12b.


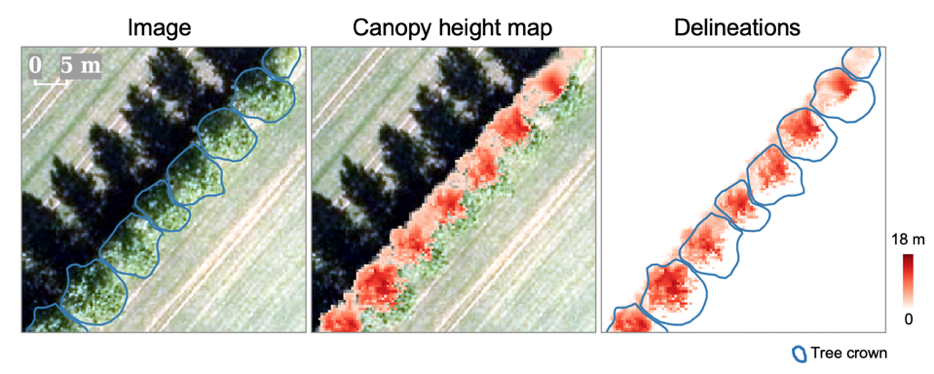


Figure S17. **Spatial mismatch between the aerial images and the LiDAR derived canopy height maps.**

Table S3. Performance comparison between separate models trained using different between-crown gap penalty weights. Evaluation on the whole testing data. Here, the tree count was derived by enumerating the segmented individual tree crowns.

| Model | Gap penalty | F1-score | Relative bias for counts (%) | R^2^ for counts | Relative bias for crown areas (%) |
| --- | --- | --- | --- | --- | --- |
| G1 | 1 | 0.79 | -16.5 | 0.93 | -16.6 |
| G2 | 3 | 0.77 | -10.7 | 0.91 | -19.9 |
| G3 | 5 | 0.75 | -9.6 | 0.92 | -24.0 |
| G4 | 7 | 0.75 | -5.3 | 0.96 | -24.9 |
| G5 | 10 | 0.73 | -7.9 | 0.97 | -27.5 |


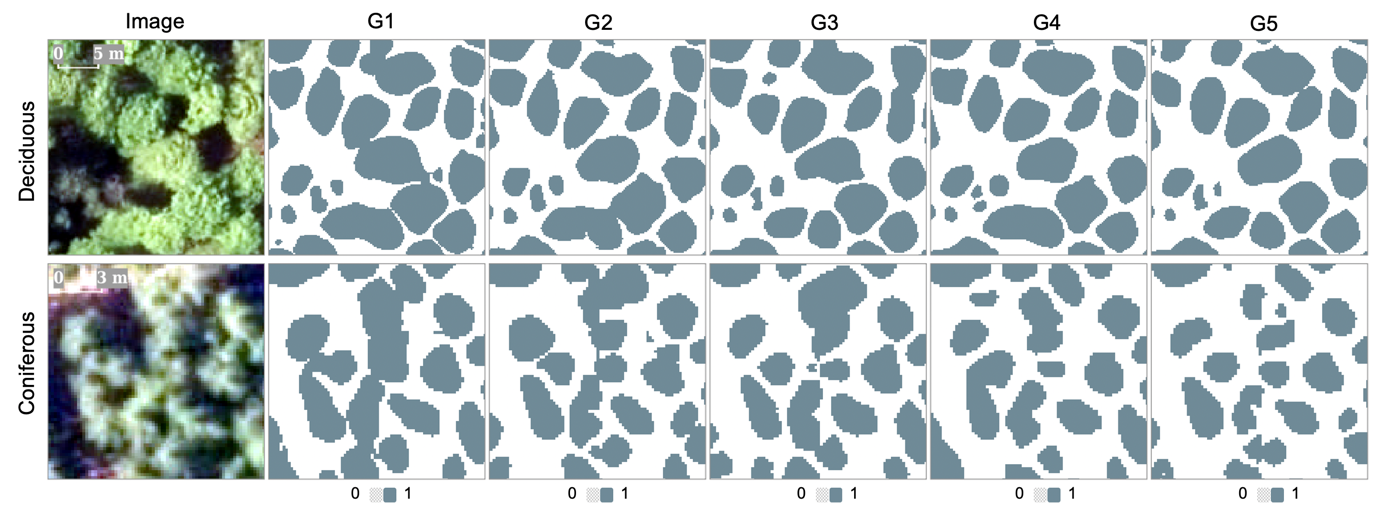


Figure S18. **Performance comparison between separate models trained using different between-crown gap penalty weights.**

Table S4. Performance comparison between separate models trained using different Gaussian kernel parameters, including kernel size and sigma. Evaluations on the whole testing data.

| Model | Kernel size | Sigma | F1-score | Relative bias for counts (%) | R^2^ for counts | Relative bias for crown areas (%) |
| --- | --- | --- | --- | --- | --- | --- |
| K1 | 9 | 3 | 0.77 | 46.8 | 0.90 | -21.5 |
| K2 | 11 | 3.5 | 0.77 | 25.1 | 0.93 | -21.2 |
| K3 | 13 | 4 | 0.77 | 19.9 | 0.94 | -19.6 |
| K4 | 15 | 4 | 0.78 | 10.3 | 0.93 | -22.6 |


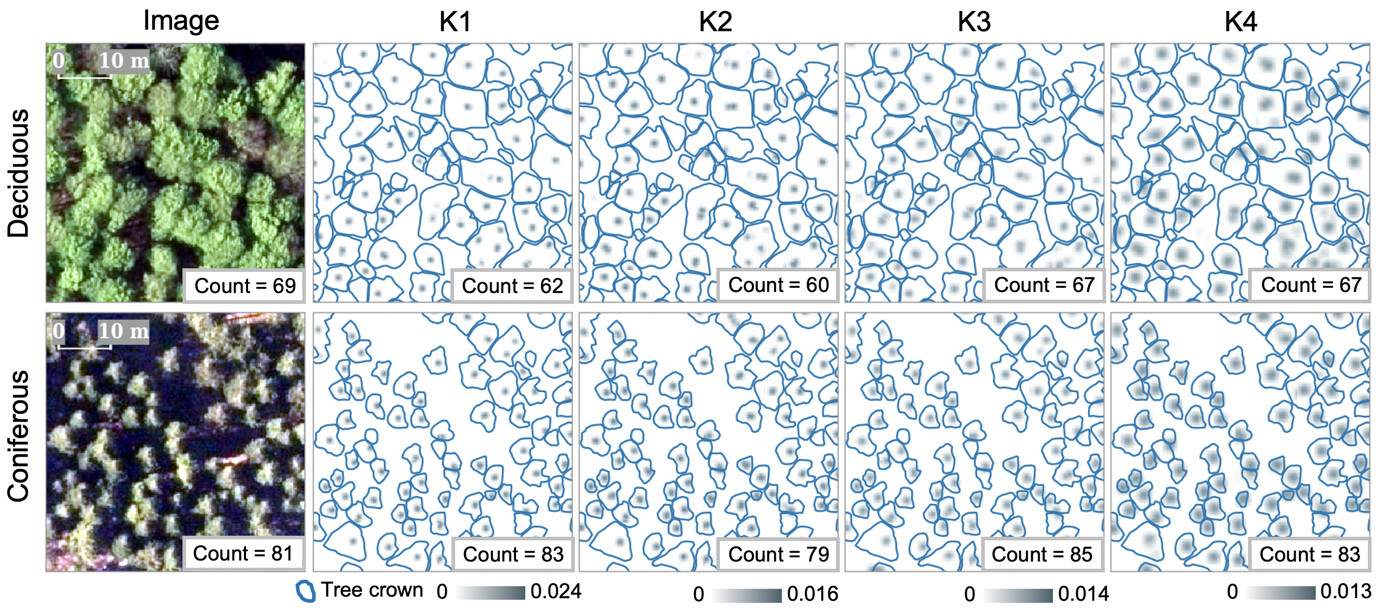


Figure S19. **Performance comparison between separate models trained using different Gaussian kernel parameters, including kernel size and sigma.**

Table S5. Performance comparison between the multi-task model (MT) and the single-task model (ST). Evaluation on the whole testing data. The multi-task model contained two output branches designed for the counting and segmentation tasks, respectively. The single-task model produced only the crown segmentation results which, by post-processing, yielded the tree counts.

| Model | F1-score | Relative bias for counts (%) | Overall bias for counts (%) | R^2^ for counts | Relative bias for crown areas (%) |
| --- | --- | --- | --- | --- | --- |
| MT | 0.76 | 10.3 | 2.0 | 0.93 | -22.6 |
| ST | 0.75 | -9.6 | 10.9 | 0.92 | -24.0 |


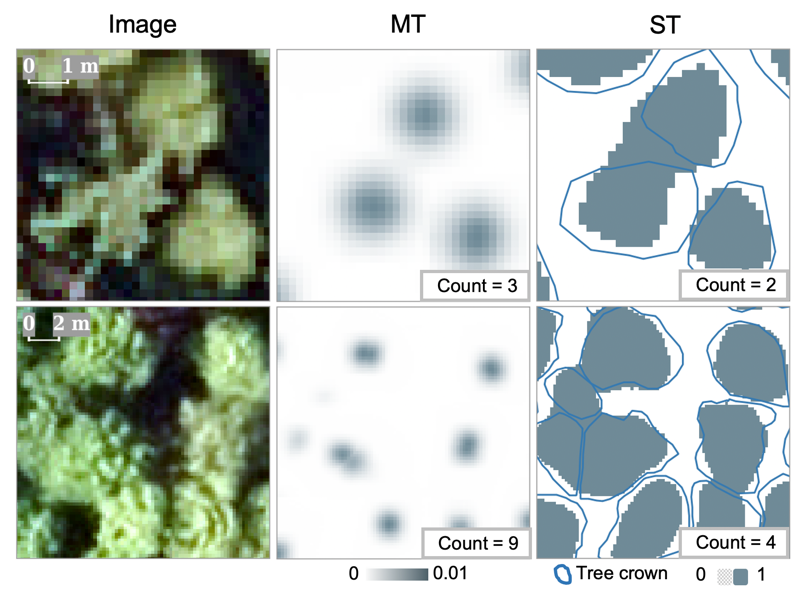


Figure S20. **Performance comparison between the multi-task model (MT) and the single-task model (ST).** Evaluation on the whole testing data. The multi-task model contained two output branches designed for the counting and segmentation tasks, respectively. The single-task model produced only the crown segmentation results which, by post-processing, yielded the tree counts.


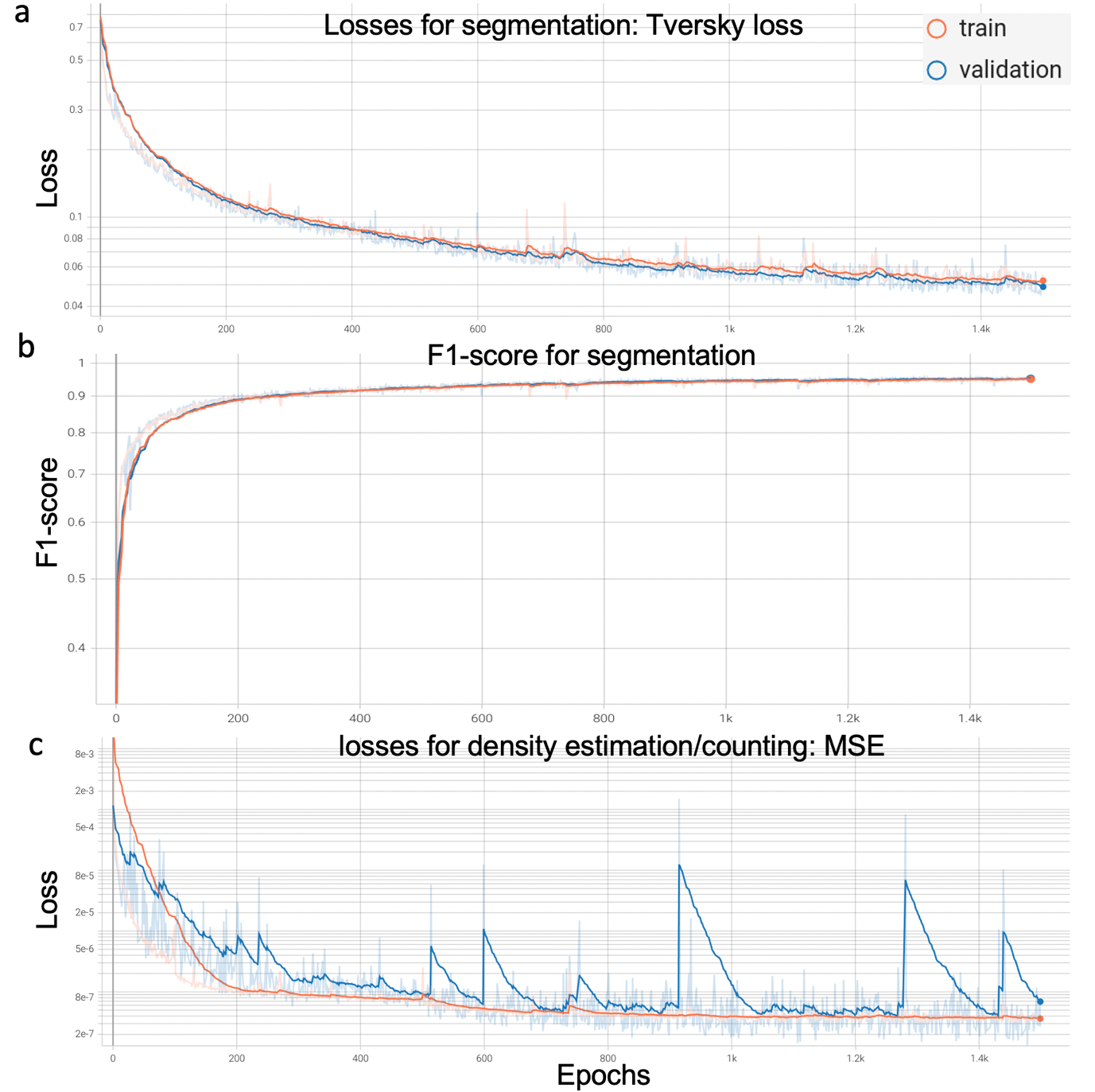


Figure S21. **Training and validation curves for the counting and crown segmentation model. a,** The loss for training the segmentation branch (Tversky loss). **b,** The F1-score used as a monitoring metric during training. **c**, The loss for training the counting/density estimation branch (MSE loss).


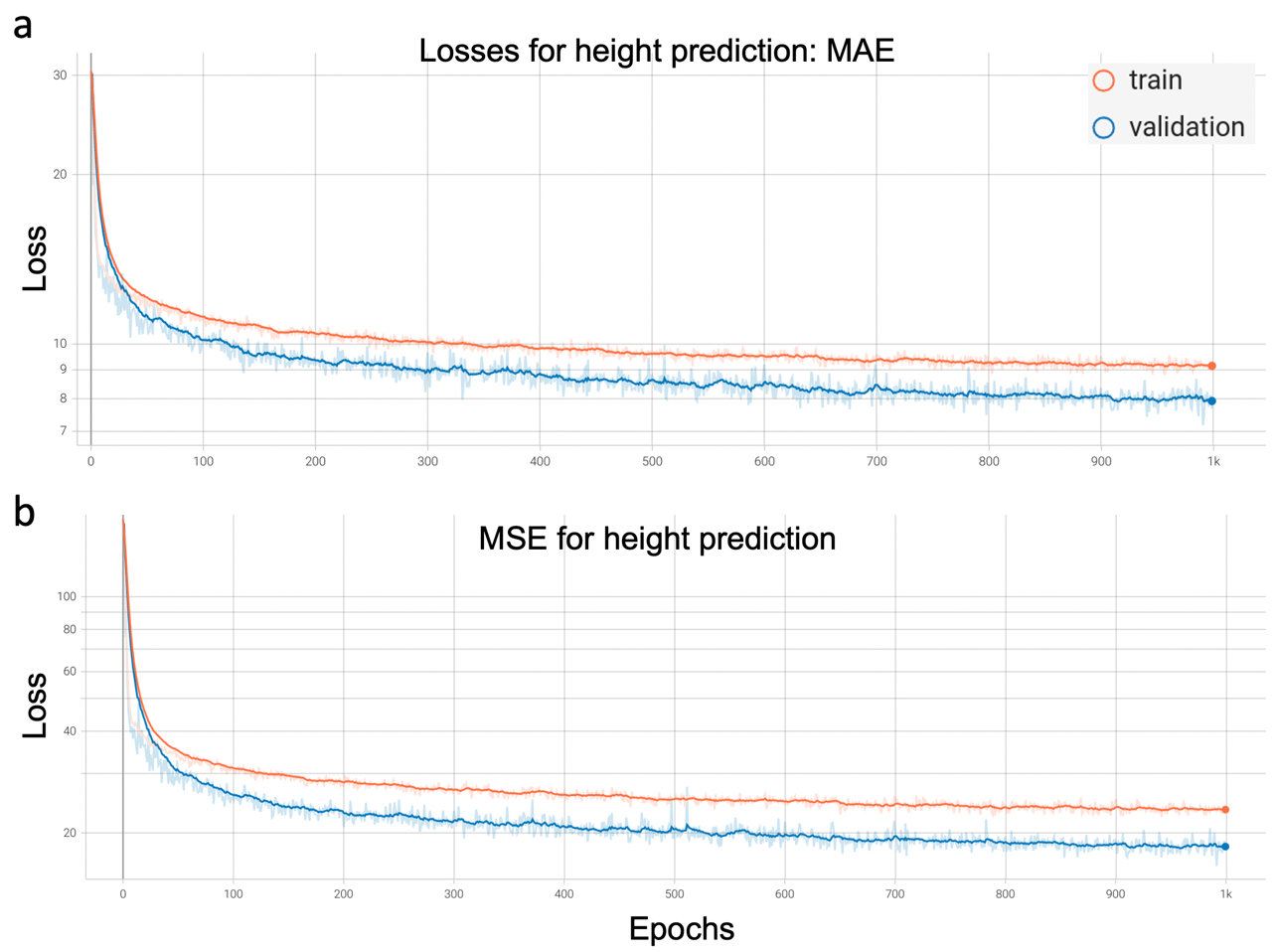


Figure S22. **Training and validation curves for the height prediction model. a,** The loss for training the height prediction model (MAE loss). **b,** The MSE loss used as a monitoring metric during training.


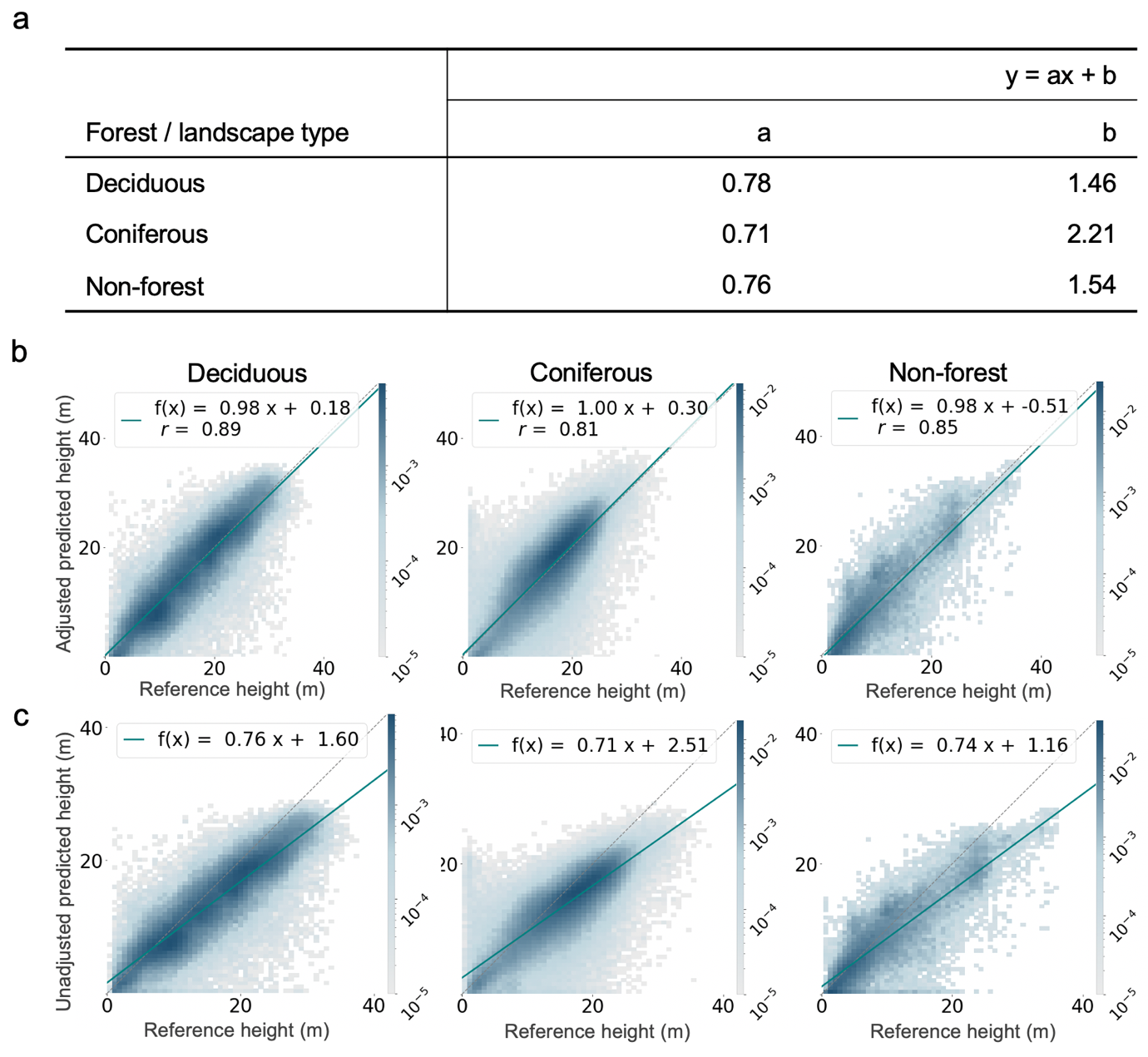


Figure S23. **Adjusting parameters for the tree height prediction model using linear regression. a,** Linear regression (y = ax + b) fitted on the predicted height (y) and the reference height (x) for deciduous, coniferous, and non-forest trees, respectively, using the validation data. **b,** Evaluated on the standalone test data: adjusted predicted height against reference height for three forest / landscape types. c, Evaluated on the standalone test data: unadjusted height against reference height.


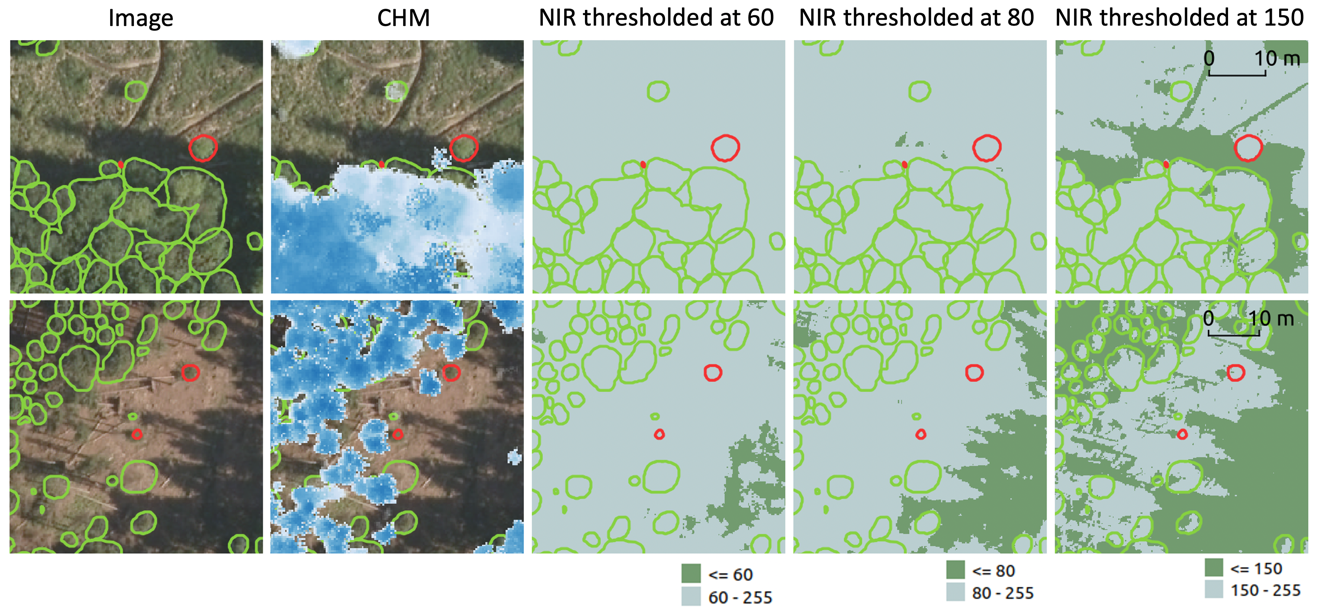


Figure S24. **Removal of suspicious tree height references based on the NIR values and the height values.** All detected tree crowns are shown by the polygons (in green and in red). An expansion factor was applied to the original crown segmentations. The red ones were removed since the height values (< 1 m) were likely to be incorrect due to the shift of the dataset. The threshold of NIR could take a wide range of values and the specific value was largely affected by the stretching and normalization of the entire dataset. We noticed that lower thresholds (60) tended to reduce distinction between vegetation and non-vegetation. While with higher values (150), the risk of missing trees with low spectral values increased. Generally, medium-to-low thresholds could ensure that trees of various spectral properties were included. We found that a moderate value such as 80 could properly differentiate vegetation from non-vegetation and was able to help remove trees with suspicious LiDAR heights.


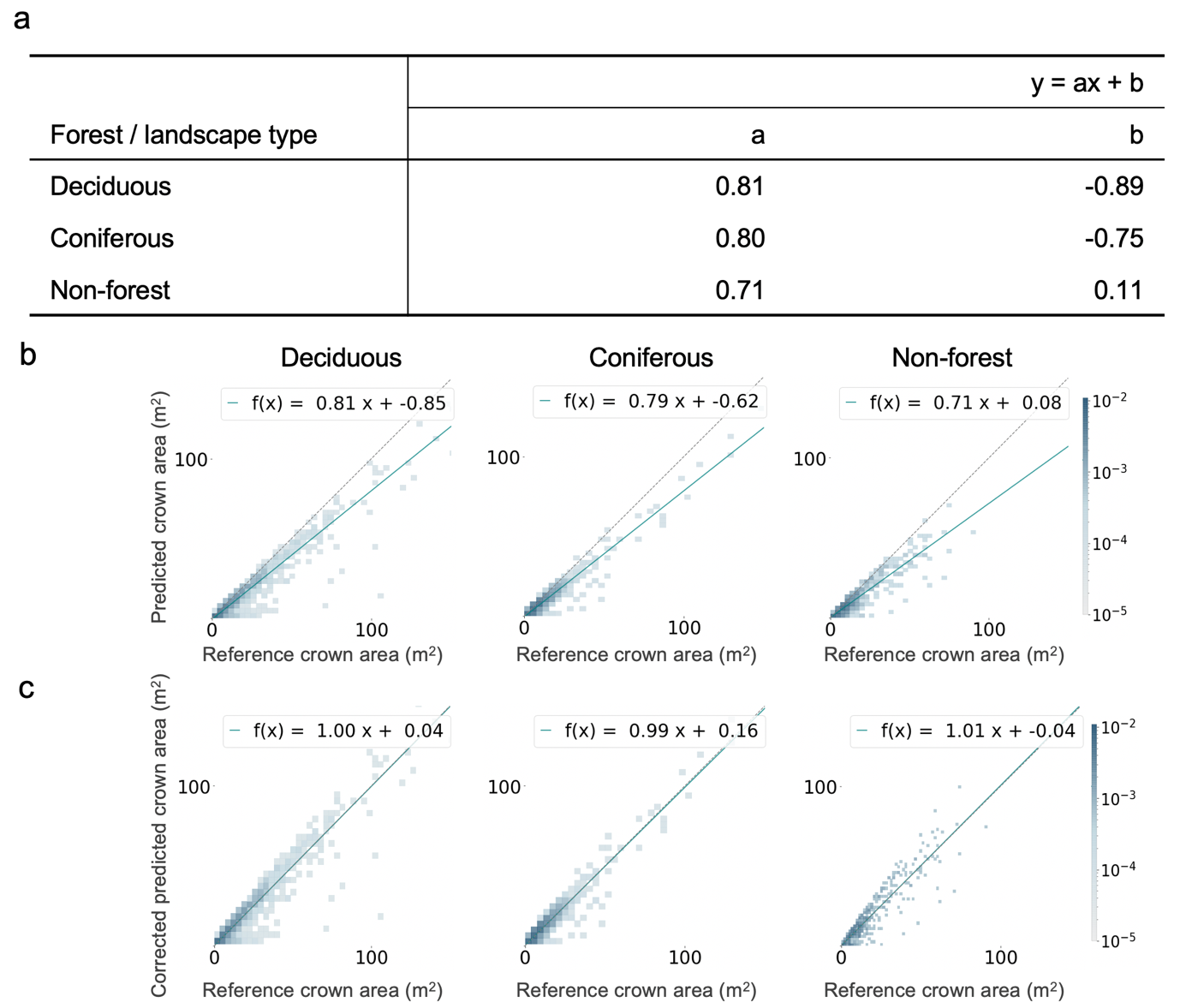


Figure S25. **Correction of tree crown area predictions using linear regression. a,** Linear regression (y = ax + b) fitted on the predicted crown area (y) and the reference crown area (x) for deciduous, coniferous, and non-forest trees, respectively. **b,** Evaluated on the standalone test data: predicted crown area (without correction) against reference crown area for three forest / landscape types, which is the same as Figure 3c. **c,** Evaluated on the standalone test data: corrected crown area against reference crown area.

Table S6. **Neural network architecture of the tree counting and crown segmentation model.**

| **Model 1: Tree counting and crown segmentation** | | | | | |
| --- | --- | --- | --- | --- | --- |
| Layer No. | Layer details | Output shape | Layer No. | Layer details | Output shape |
| L0 | Input | (256, 256, D) | L15 | Self-attention Concat (L14, L11) | (32, 32, 1536) |
| L1 | Conv2d (64, 3) + Relu | (256, 256, 64) | L16 | Conv2d (512, 3) + Relu | (32, 32, 512) |
| L2 | Conv2d (64, 3) + Relu + BN | (256, 256, 64) | L17 | Conv2d (512, 3) + Relu + BN | (32, 32, 512) |
| L3 | MaxPooling2d | (128, 128, 64) | L18 | Self-attention Concat (L17, L8) | (64, 64, 768) |
| L4 | Conv2d (128, 3) + Relu | (128, 128, 128) | L19 | Conv2d (256, 3) + Relu | (64, 64, 256) |
| L5 | Conv2d (128, 3) + Relu + BN | (128, 128, 128) | L20 | Conv2d (256, 3) + Relu + BN | (64, 64, 256) |
| L6 | MaxPooling2d | (64, 64, 128) | L21 | Self-attention Concat (L20, L5) | (128, 128, 384) |
| L7 | Conv2d (256, 3) + Relu | (64, 64, 256) | L22 | Conv2d (128, 3) + Relu | (128, 128, 128) |
| L8 | Conv2d (256, 3) + Relu + BN | (64, 64, 256) | L23 | Conv2d (128, 3) + Relu + BN | (128, 128, 128) |
| L9 | MaxPooling2d | (32, 32, 256) | L24 | Self-attention Concat (L23, L2) | (256, 256, 192) |
| L10 | Conv2d (512, 3) + Relu | (32, 32, 512) | L25 | Conv2d (64, 3) + Relu | (256, 256, 64) |
| L11 | Conv2d (512, 3) + Relu + BN | (32, 32, 512) | L26 | Conv2d (64, 3) + Relu + BN | (256, 256, 64) |
| L12 | MaxPooling2d | (16, 16, 512) | L27A | Conv2d (1, 1) + Linear | (256, 256, 1) |
| L13 | Conv2d (1024, 3) + Relu | (16, 16, 1024) | L27B | Conv2d (1, 1) + Linear | (256, 256, 1) |
| L14 | Conv2d (1024, 3) + Relu + BN | (16, 16, 1024) |  |  |  |
| # Trainable parameters: 31,909,734 | | | | | |

| **Self-attention block (e.g., L15)** | | |
| --- | --- | --- |
| Layer No. | Layer details | Output shape |
| A1 | UpSampling2d (L14) | (32, 32, 1024) |
| A2 | Conv2d (256, 1) (L11) | (32, 32, 256) |
| A3 | Conv2d (256, 1) (A1) | (32, 32, 256) |
| A4 | Add (A2, A3) + Relu | (32, 32, 256) |
| A5 | Conv2d (1, 1) (A4) + Sigmoid | (32, 32, 1) |
| A6 | Multiply (A5, L11) | (32, 32, 512) |
| A7 | Concatenate (A6, A1) | (32, 32, 1536) |

Table S7. **Neural network architecture of the tree counting and crown segmentation model with multi-resolution input layers.**

| **Model 1v (Multi-resolution inputs): Tree counting and crown segmentation** | | | | | |
| --- | --- | --- | --- | --- | --- |
| Layer No. | Layer details | Output shape | Layer No. | Layer details | Output shape |
| M0-0 | Input 1 | (256, 256, D1) | M17 | Conv2d (1024, 3) + Relu | (16, 16, 1024) |
| M0-1 | Conv2d (64, 3) + Relu | (256, 256, 64) | M18 | Conv2d (1024, 3) + Relu + BN | (16, 16, 1024) |
| M0-2 | Conv2d (64, 3) + Relu + BN | (256, 256, 64) | M19 | Self-attention Concat (M18, M15) | (32, 32, 1536) |
| M0-3 | MaxPooling2d | (128, 128, 64) | M20 | Conv2d (512, 3) + Relu | (32, 32, 512) |
| M1-0 | Input 2 | (128, 128, D2) | M21 | Conv2d (512, 3) + Relu + BN | (32, 32, 512) |
| M1-1 | Conv2d (64, 3) + Relu | (128, 128, 64) | M22 | Self-attention Concat (M21, M12) | (64, 64, 768) |
| M1-2 | Conv2d (64, 3) + Relu + BN | (128, 128, 64) | M23 | Conv2d (256, 3) + Relu | (64, 64, 256) |
| M7 | Concatenate (M0-3, M1-2) | (128, 128, 128) | M24 | Conv2d (256, 3) + Relu + BN | (64, 64, 256) |
| M8 | Conv2d (128, 3) + Relu | (128, 128, 128) | M25 | Self-attention Concat (M24, M9) | (128, 128, 384) |
| M9 | Conv2d (128, 3) + Relu + BN | (128, 128, 128) | M26 | Conv2d (128, 3) + Relu | (128, 128, 128) |
| M10 | MaxPooling2d | (64, 64, 128) | M27 | Conv2d (128, 3) + Relu + BN | (128, 128, 128) |
| M11 | Conv2d (256, 3) + Relu | (64, 64, 256) | M28 | Self-attention Concat (M27, M0-3) | (256, 256, 192) |
| M12 | Conv2d (256, 3) + Relu + BN | (64, 64, 256) | M29 | Conv2d (64, 3) + Relu | (256, 256, 64) |
| M13 | MaxPooling2d | (32, 32, 256) | M30 | Conv2d (64, 3) + Relu + BN | (256, 256, 64) |
| M14 | Conv2d (512, 3) + Relu | (32, 32, 512) | M31A | Conv2d (1, 1) + Linear | (256, 256, 1) |
| M15 | Conv2d (512, 3) + Relu + BN | (32, 32, 512) | M31B | Conv2d (1, 1) + Linear | (256, 256, 1) |
| M16 | MaxPooling2d | (16, 16, 512) |  |  |  |
| # Trainable parameters: 32,021,158 | | | | | |

Table S8. **Neural network architecture of the canopy height prediction model.**

| **Model 2: Canopy height prediction** | | | | | |
| --- | --- | --- | --- | --- | --- |
| Layer No. | Layer details | Output shape | Layer No. | Layer details | Output shape |
| L0 | Input | (256, 256, D) | L13 | Conv2d (1024, 3) + Relu | (16, 16, 1024) |
| L1 | Conv2d (64, 3) + Relu | (256, 256, 64) | L14 | Conv2d (1024, 3) + Relu + BN | (16, 16, 1024) |
| L2 | Conv2d (64, 3) + Relu + BN | (256, 256, 64) | L15 | Self-attention Concat (L14, L11) | (32, 32, 1536) |
| L3 | MaxPooling2d | (128, 128, 64) | L16 | Conv2d (512, 3) + Relu | (32, 32, 512) |
| L4 | Conv2d (128, 3) + Relu | (128, 128, 128) | L17 | Conv2d (512, 3) + Relu + BN | (32, 32, 512) |
| L5 | Conv2d (128, 3) + Relu + BN | (128, 128, 128) | L18 | Self-attention Concat (L17, L8) | (64, 64, 768) |
| L6 | MaxPooling2d | (64, 64, 128) | L19 | Conv2d (256, 3) + Relu | (64, 64, 256) |
| L7 | Conv2d (256, 3) + Relu | (64, 64, 256) | L20 | Conv2d (256, 3) + Relu + BN | (64, 64, 256) |
| L8 | Conv2d (256, 3) + Relu + BN | (64, 64, 256) | L21 | Self-attention Concat (L20, L5) | (128, 128, 384) |
| L9 | MaxPooling2d | (32, 32, 256) | L22 | Conv2d (128, 3) + Relu | (128, 128, 128) |
| L10 | Conv2d (512, 3) + Relu | (32, 32, 512) | L23 | Conv2d (128, 3) + Relu + BN | (128, 128, 128) |
| L11 | Conv2d (512, 3) + Relu + BN | (32, 32, 512) | L24 | Conv2d (1, 1) + Linear | (128, 128, 1) |
| L12 | MaxPooling2d | (16, 16, 512) |  |  |  |
| # Trainable parameters: 31,755,780 | | | | | |

Table S9. **Data split and usage for all models in this study.**

| **Dataset** | **Usage** |
| --- | --- |
| Training | Training of model parameters |
| Validation | Model selection and hyper-parameter tuning |
| Testing | Evaluation (shown in Results) |

**References**

1. European Environment Agency (EEA). Copernicus Land Monitoring Service <2018>.

2. Hansen, M. C. *et al.* High-Resolution Global Maps of 21st-Century Forest Cover Change. *Science (1979)* **342**, (2013).
